# Comparative Gut Microbiome Alterations in Myalgic Encephalomyelitis/Chronic Fatigue Syndrome and Long COVID-19 Syndrome

**DOI:** 10.3390/biomedicines14061183

**Published:** 2026-05-22

**Authors:** Deyan Donchev, Ralitsa Nikolova, Katya Vaseva, Hristo Taskov, Mariana Murdjeva, Michael Maes, Ivan Nikolaev Ivanov

**Affiliations:** 1 National Reference Laboratory for Control and Monitoring of Antimicrobial Resistance, Department of Microbiology, National Center of Infectious and Parasitic Diseases, 26 Yanko Sakazov Blvd., 1504 Sofia, Bulgaria; 2 Strategic Research and Innovation Program for the Development of Medical University of Plovdiv (SRIPD-MUP), 4002 Plovdiv, Bulgaria; 3 Department of Medical Microbiology and Immunology-“Prof. Dr. Elissay Yanev”, Faculty of Medicine, Medical University of Plovdiv, 4002 Plovdiv, Bulgaria; 4 Research Institute at Medical University of Plovdiv, 4002 Plovdiv, Bulgaria; 5 Laboratory of Microbiology, University Hospital St. George, 4002 Plovdiv, Bulgaria; 6 Department of Microbiology and Virology, Medical University of Pleven, 5800 Pleven, Bulgaria; 7 Institute of Innovation and Smart Technology, University of Telecommunication and Posts, 1700 Sofia, Bulgaria; 8 Sichuan Provincial Center for Mental Health, Sichuan Provincial People’s Hospital, School of Medicine, University of Electronic Science and Technology of China, Chengdu 610072, China; 9 Key Laboratory of Psychosomatic Medicine, Chinese Academy of Medical Sciences, Chengdu 610072, China

**Keywords:** gut microbiome composition, dysbiosis, myalgic encephalomyelitis (ME), chronic fatigue syndrome (CFS), long COVID (LC), post-acute COVID-19 Syndrome (PACS), 16S amplicon sequencing

## Abstract

**Background:** Myalgic encephalomyelitis/chronic fatigue syndrome (ME/CFS) and long COVID-19 syndrome (LC) show substantial clinical overlap, but direct comparative microbiome studies remain limited. **Methods:** In this cross-sectional study, we compared the fecal gut microbiome of patients with ME/CFS, LC, and healthy controls (HC) within a unified analytical framework using 16S rRNA profiling, differential abundance testing, and multivariate modeling. We also examined associations between microbiome variation and questionnaire-derived symptom-domain scores. **Results:** Alpha-diversity did not differ significantly among groups, whereas beta-diversity analyses showed small but significant disease-associated community differences with broad overlap between cohorts. Differential abundance analysis identified stronger signals in disease-versus-control contrasts than in the direct ME/CFS vs. LC contrast. Both ME/CFS and LC shared enrichment of *Sutterella* and depletion of *Terrisporobacter* and *Lachnospiraceae* relative to HC. Predicted functional profiling showed shared disease-versus-control changes in pathways related to anaerobic acetate/H_2_ carbon flow, inositol/polyol degradation, phosphonate/C1-related metabolism, and lysine-derived fermentation. Regression analyses showed the strongest microbiome associations with fatigue-related and physiosomatic domains, while affective, cognitive, and gastrointestinal outcomes showed weaker signals. **Conclusions:** Overall, these findings support the presence of overlapping but non-identical gut microbiome alterations in ME/CFS and LC. The results provide a basis for future longitudinal and multi-omics studies aimed at clarifying the stability, functional relevance, and clinical utility of these microbial patterns.

## 1. Introduction

Soon after the onset of the COVID-19 pandemic, accumulating evidence indicated that some patients continue to feel ill months to years after they have recovered from the initial COVID-19 infection [1]. They may experience a variety of symptoms, which commonly include extreme fatigue, “brain fog”, shortness of breath, chest pain, heart palpitations, sleep dysfunction, depression, anxiety, and muscle and joint pain that can severely limit daily functioning [2]. This condition, often called “long COVID syndrome” (LC) or “post-acute COVID sequelae”, shares many symptoms with myalgic encephalomyelitis/chronic fatigue syndrome (ME/CFS), suggesting the two illnesses may have similar underlying biological abnormalities [3].

ME/CFS has an estimated prevalence of approximately 0.89%, corresponding to tens of millions of affected individuals worldwide, and is associated with markedly reduced health-related quality of life [4,5]. LC has been estimated to affect 6.2% of individuals after symptomatic SARS-CoV-2 infection at 3 months and is likewise associated with persistent work-related impairment and occupational disruption in a substantial proportion of patients [6,7]. Given the documented burden of both conditions, a better understanding of their pathophysiological mechanisms is a clinical and public health priority.

Over the past decade, the gut microbiome has emerged as a major regulator of immune homeostasis, metabolic function, and neurological processes, with evidence implicating gut dysbiosis in a broad spectrum of systemic and neuropsychiatric diseases [8]. Changes in gut microbiota composition in both LC and ME/CFS, along with the frequent gastrointestinal symptoms reported in these patients, suggest a potential role for gut dysbiosis in the pathogenesis of both conditions [9].

Recent studies have reported evidence of gut microbiome imbalance in ME/CFS patients, characterized by reduced microbial diversity and shifts in the relative abundance of harmful and beneficial bacteria, especially those engaged in short chain fatty acids (SCFAs) synthesis [10,11]. Researchers noted a reduction in *Firmicutes* and an increase in *Bacteroidetes*, together with lower abundance of key commensals with anti-inflammatory functions such as *Faecalibacterium*, *Roseburia*, *Bifidobacterium*, *Eubacterium rectale*, and *Faecalibacterium prausnitzii* [10,11,12,13,14,15,16,17]. At the same time, several taxa are enriched in ME/CFS and are thought to contribute to a more proinflammatory microbial profile. These include *Alistipes*, *Coprobacillus*, *Eggerthella*, and *Blautia* [15,18,19]. Similarly, persistent alterations in the gut microbiome have been described in patients with LC, raising the possibility that microbiome perturbation may contribute to its ongoing symptoms [20]. Among the most frequently reported changes are an increased relative abundance of *Bacteroides* and *Flavonifractor* and a decrease in *Bifidobacterium* and *Dorea* [21,22,23,24,25]. At the species level, *Ruminococcus gnavus* has been consistently associated with a higher likelihood of LC across multiple studies, whereas the SCFAs producer *Faecalibacterium prausnitzii* has shown a consistent inverse association [26,27,28,29].

Accumulating evidence indicates that the gut microbiome may modulate cognition and behavior through the gut–brain axis, with alterations in microbial composition being associated with depressive-like behaviors [30]. Recent studies support the integration of gut microbiome profiling with validated symptom questionnaires in both LC and ME/CFS. In LC, Zhang et al. showed that persistent symptoms were associated with poorer quality of life, higher anxiety and depression scores, reduced gut microbial diversity, and depletion of SCFA-producing genera [21]. Smaller LC interventional studies have similarly linked gut microbiome analysis with multidomain clinical measures, suggesting that changes in symptom burden may parallel microbiome and immune alterations [31]. In acute hospitalized COVID-19, characteristic clustering of taxa such as *Phocaeicola*, *Bacteroides*, and *Mitsuokella* have been linked to greater somatic or neuropsychiatric scores [32]. In ME/CFS, the literature is more limited but still provides precedent: Rao et al. showed that stool microbial changes during probiotic treatment were accompanied by reduced Beck Anxiety Inventory scores, while newer microbiome-targeted trials in ME/CFS have increasingly incorporated self-reporting tools about fatigue, depression/anxiety [33]. Together, these studies justify analyzing microbiome composition in parallel with questionnaire-derived domains of neuropsychiatric symptoms and quality of life in patients, thus highlighting that dysbiosis may have an important role in affecting emotion and behavior. 

Gut microbiome dysbiosis in both ME/CFS and LC is thought to promote a persistent state of low-grade immune activation and impaired neuronal health through the disruption of the gut–blood barrier and gut–brain axis [34,35]. The increased intestinal permeability, known as “leaky gut,” may allow the translocation of inflammatory microbial products, including lipopolysaccharides, into the circulation, thereby contributing to systemic inflammation and disease severity [36,37]. However, interpretation of the reported changes in gut microbiome composition in these illnesses remains challenging. Owing to inconsistencies in study design, inadequate control of confounding factors, and even differences in microbiome profiling methods, no consistent microbial alterations or definitive disease-specific signatures have yet been identified in either ME/CFS or LC [38,39,40,41].

Despite the growing literature on gut dysbiosis in both ME/CFS and LC, direct comparisons of gut microbiome changes in these illnesses remain limited. It is still unclear whether the two conditions share common microbial features or show distinct disease-specific patterns, and if these changes are linked to symptoms of depression and anxiety. In this study, we compared the fecal gut microbiome of patients with ME/CFS, LC, and healthy controls to seek for shared or distinct microbial features and to examine their association with depression and anxiety symptoms.

## 2. Materials and Methods

### 2.1. Study Design and Characteristics of Study Population

This cross-sectional case–control study included 163 participants: 76 patients with ME/CFS (17 males and 59 females), 50 patients with LC condition (13 males and 37 females), and 37 HC (11 males and 26 females). Participants were recruited at two centers in Bulgaria and provided written informed consent prior to inclusion. Eligibility required age between 18 and 65 years.

Enrollment of both ME/CFS and LC patients was based on established clinical criteria. Patients with ME/CFS were selected according to the 2015 Institute of Medicine/National Academy of Medicine diagnostic criteria [42], requiring core ME/CFS symptoms for at least 6 months with no alternative diagnosis. Patients with LC were recruited according to the WHO clinical case definition [43], requiring documented SARS-CoV-2 infection and persistent or newly emerging symptoms 3 months after acute COVID-19, lasting for at least 2 months, and not explained by another diagnosis. Standardized self-reported and clinician-rated instruments were subsequently used to quantify symptom burden and derive symptom-domain variables for phenotype-related analyses. HC were recruited from the Medical University of Plovdiv and the National Center of Infectious and Parasitic Diseases in Sofia. They were required to have no history of ME/CFS, LC condition, or other chronic symptom complexes compatible with these diagnoses, no current acute infection, and no history of positive SARS-CoV-2 PCR test or detectable anti-SARS-CoV-2 IgG. Eligibility was assessed through a detailed interview conducted by a team of specialists in immunology and microbiology.

Participants were excluded if they had a current or past diagnosis of major psychiatric disorder or substance use disorder, except tobacco use disorder. Additional exclusions included cancer, renal disease, liver disease, neurodegenerative disorders, systemic autoimmune disease, pregnancy or breastfeeding, and recent use of antibiotics, immunosuppressants, corticosteroids, or immune-modulating medications within the previous 3 months.

The research was conducted in accordance with the Declaration of Helsinki and was approved by the institutional ethics board of Medical University of Plovdiv (Protocol No. 2, 7 February 2024), for studies involving humans.

### 2.2. Clinical Measurements

Demographic and baseline clinical data collected at the study visit included age, sex, height and weight, body mass index (BMI), education, employment status, smoking status, pets, and relevant medical history. Participants completed standardized symptom instruments including the FibroFatigue scale (FF), Hamilton Anxiety Rating Scale (HAMA), Hamilton Depression Rating Scale (HAMD), DePaul Symptom Questionnaire, WHOQOL-BREF, and Childhood Trauma Questionnaire (CTQ). In addition to total questionnaire scores, derived symptom-domain and composite variables were generated for downstream phenotype screening, including pure fatigue, physiosomatic, autonomic, gastrointestinal, cognitive, sleep, or phenome-related composites.

### 2.3. Sample Collection and Processing

Participants were given written and verbal instructions to ensure the proper collection, avoiding contamination with urine or water. Fecal samples were self-collected by participants in sterile containers and delivered to designated personnel within one hour. Upon arrival, the samples were aliquoted (100 mg of fecal material per aliquot) in sterile cryovials and DNA/RNA shield (Zymo Research, Irvine, CA, USA, #R1200-125). The aliquots were kept at −80 °C until DNA extraction.

### 2.4. Stool DNA Extraction

DNA was extracted with the Zymo Quick-DNA HMW MagBead Kit (Zymo Research, Irvine, CA, USA, #D6060) following the manufacturer’s instructions for samples in DNA/RNA Shield, with a modified mechanical lysis step. In brief, 100 mg fecal aliquots were mixed with 250 µL 2× DNA/RNA Shield, 150 µL nuclease-free water, in 0.5 mL screw-cap tubes prefilled with approximately 100 µL 0.1/0.5 mm zirconia beads mix. Bead-beating was performed at maximum speed for 13 min on a Vortex-genie-2 (Scientific Industries Inc. 2021, Bohemia, NY, USA, # SI-0236) equipped with a horizontal adapter followed by centrifugation at 15,000× *g* for 2 min. The clarified supernatant was transferred to tubes containing 20 µL Proteinase K. DNA yield and purity (A260/280 and A260/230 ratios) were measured and recorded.

### 2.5. Sequencing and Reads Processing

Complete 16S rRNA gene libraries were prepared using the Microbial Amplicon Barcoding Kit 24 V14 (Oxford Nanopore Technologies, Oxford, UK, #SQK-MAB114.24) sequenced in no specific order in batches on MinION Mk1b (Oxford Nanopore Technologies, Oxford, UK, # MIN-101B). Additionally, ZymoBIOMICS Microbial Community DNA Standard (Zymo Research, Irvine, CA, USA, #D6305) was also included to assess bias introduced from library preparation. Negative extraction controls and no-template PCR controls were not included in the present workflow.

Raw signal data were basecalled with sup model 5.2.0 in Dorado v1.3.1. During sequencing in MinKNOW v25.09.16, barcode trimming was disabled so that barcode sequences were retained. Reads were then demultiplexed using Barbell v0.3.0 and filtered with chopper v0.12.0b to retain sequences with a minimum mean Q-score of 15 and a minimum length of 800 bp.

### 2.6. Taxonomic Assignment and Microbiome Data Preprocessing

Taxonomic assignment was performed using EMU v3.5.5 against the GreenGenes2 2024.09 16S reference database. The resulting tables (by default filtered at 0.0001) were merged and imported into QIIME2-compatible feature and taxonomy tables and used for differential abundance analysis (DAA). In parallel, amplicon reads were processed with NanoASV v1.2.2, and MetaCyc pathway profiles were inferred from the resulting taxonomic data using PICRUSt2 v2.6.2. The resulting pathway tables were subjected to DAA with MaAsLin3 v1.1.2 as explained in 2.7.1.

### 2.7. Bioinformatic and Statistical Analysis

Initial microbial diversity, alpha- and beta-diversity using Shannon index, Pielou’s evenness, Bray–Curtis, and Jaccard were performed in QIIME2 v2025.10. Sample-level structure was explored by PCA and t-SNE, and group differences in community composition were evaluated from Bray–Curtis distances using ordination, PERMANOVA, and dispersion testing. PCA was performed in base R, t-SNE with Rtsne v0.17, and distance-based analyses with vegan v2.7.3.

#### 2.7.1. Differential Abundance Analysis

DAA was conducted in parallel with ANCOM-BC2 v2.12.0 and MaAsLin3 v1.1.2 with default parameters at both genus and species levels with tables with 10% prevalence. For each taxonomic rank and model type, all three groups were included in one model, and the pairwise contrasts were extracted from that shared model rather than performing multiple two-group analyses. This strategy was used because the study aimed to evaluate both shared and disease-specific microbiota patterns across two clinically overlapping post-infectious syndromes, while keeping the covariate adjustment and model structure consistent across all contrasts. Both DA tools were used with CLR-normalized tables with sex, BMI, patient group and age as adjustment covariates. Log fold changes, standard errors, and multiple-testing-adjusted significance values were reported. Structural zeros were also assessed. Statistical significance was defined as q < 0.05, while *p* < 0.05 was retained for exploratory interpretation where appropriate.

#### 2.7.2. Multivariate Modeling

Multivariate modeling was performed on the CLR-transformed genus-level abundance table using glmnet v4.1.10 and mixOmics v6.34.0. Pairwise group classification/discrimination was performed for HC vs. ME/CFS, HC vs. LC, and ME/CFS vs. LC. In these models, patient_group was used as the outcome and was therefore not included as a covariate. Adjusted models included age, BMI, and sex. Residualization was applied for mixOmics. glmnet v4.1.10 was used for pairwise classification and continuous-score regression. Models were evaluated by repeated 5-fold cross-validation with 20 repeats. Both predictive and sparse branches were examined, with lambda.1se used as the primary conservative solution and lambda.min as an exploratory solution. Classification performance was summarized by ROC AUC and balanced accuracy. mixOmics v6.34.0 was used for PLS-DA/sPLS-DA discrimination and PLS/sPLS regression. Models were evaluated by repeated 5-fold cross-validation with 10 repeats, and adjusted pairwise discrimination models were additionally assessed by permutation testing. Discrimination performance was summarized by balanced error rate (BER), and regression performance by R^2^ and RMSE.

## 3. Results

### 3.1. Cohort and Sequencing Overview

After importing the EMU-derived abundance tables into QIIME 2, the final feature table contained 163 samples and 1034 unique features, with 21,456,085 total observations. After exclusion of one low-depth outlier sample (2.3K reads), sequencing depth per sample ranged from 28,734 to 263,131 reads (mean 131,632.4; median 131,603; IQR 103,570–162,832) as shown in Supplementary Figure S1A. Alpha rarefaction analysis based on observed features showed that all samples approached a plateau well below the selected rarefaction depth, and the curves remained largely stable, indicating that the retained read depth was sufficient to capture the majority of within-sample taxonomic richness (Supplementary Figure S1B). Baseline demographic screening showed that age differed significantly across groups after FDR correction, with significant pairwise differences between HC and both disease groups, whereas weight, height, and BMI did not differ significantly. Missingness was negligible for age and approximately 12% for weight, height, and BMI (Table 1). As an internal sequencing control, the ZymoBIOMICS microbial community DNA standard yielded a Microbiome Quality Index (MIQ) score of 94/100 (root mean square error = 5.80), supporting minimal bias from library preparation and read processing and overall excellent taxonomic profiling performance.

After filtering, the final disease-comparison tables used in downstream differential abundance analyses contained 224 tested taxa. These filtered tables formed the basis for diversity summaries, taxonomic composition overviews, pairwise differential abundance testing, and exploratory multivariate modeling.

### 3.2. Taxonomic Overview

**Figure 1:**
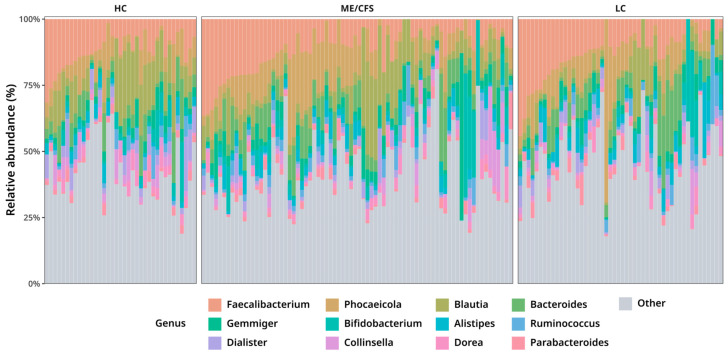
Taxa bar plots at genus level separated by cohorts. The 12 highest genera are presented and the remaining are grouped as others.

Group-wise sample-level genus composition showed that the three sample groups shared a broadly similar dominant taxonomic background, primarily composed of *Faecalibacterium*, *Phocaeicola*, *Blautia*, and *Bacteroides*, with marked inter-individual variability within each group (Figure 1). No obvious disease-specific whole-community compositional pattern was apparent from the dominant genera alone. A similar picture was observed in the supplementary phylum-level composition plot (Supplementary Figure S2), supporting the conclusion that between-group differences are subtle rather than grossly compositional.

### 3.3. Community-Level Diversity and Structure

**Figure 2:**
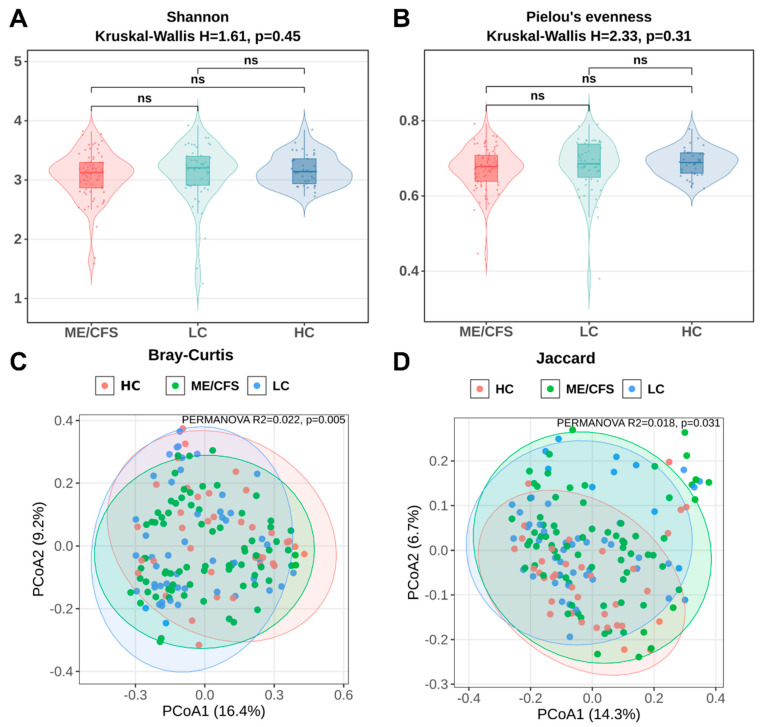
(**A**) Shannon diversity. (**B**) Pielou’s evenness. (**C**) Principal coordinates analysis based on Bray–Curtis dissimilarity, reflecting abundance-weighted community differences. (**D**) Principal coordinates analysis based on Jaccard distance, reflecting presence or absence of species. ns—not significant.

Initial diversity analyses showed no evidence of between-group differences in alpha-diversity (Figure 2). Shannon diversity did not differ across HC, ME/CFS, and LC groups (Kruskal–Wallis H = 0.66, *p* = 0.72), and the same was observed for Pielou’s evenness (H = 0.67, *p* = 0.72). In contrast, beta-diversity analyses indicated small but significant group-level differences in overall community composition. Bray–Curtis-based ordination showed a significant global group effect (PERMANOVA R^2^ = 0.019, *p* = 0.009), and a similar result was observed for Jaccard distances (R^2^ = 0.017, *p* = 0.024). Despite these significant global effects, ordination plots showed broad overlap among the three groups, indicating that community-level differences were subtle rather than cluster-defining. Descriptive PCA and t-SNE plots yielded the same overall impression of extensive overlap without discrete separation and are therefore presented as supplementary confirmation of the PCoA findings (Supplementary Figure S3).

Alpha-diversity did not differ significantly among ME/CFS, LC, and HC, indicating that the overall within-sample diversity and evenness were comparable across groups (Figure 2A,B). Beta-diversity analyses were therefore used to test whether the composition differed between groups. Bray–Curtis distance was used to capture differences in relative abundance, whereas Jaccard distance was used as a presence/absence-based measure independent of abundance. In the full cohort, PERMANOVA detected a small but significant group effect using both Bray–Curtis and Jaccard distances, indicating that the groups differed modestly in both abundance-weighted composition and presence/absence structure (Figure 2C,D; Table 2). The corresponding R^2^ values were low, showing that patient group explained only a small fraction of total community variation. For Bray–Curtis, PERMDISP was not significant, indicating that the PERMANOVA result was not driven by unequal within-group dispersion. In the complete metadata subset, the Bray–Curtis group effect remained significant after adjustment for age, BMI, and sex, supporting a disease-group association independent of these covariates. These results indicate statistically detectable, subtle and distributed community-level differences, with broad overlap among HC, ME/CFS, and LC without discrete clustering.

### 3.4. Differential Abundance and Cross-Method Convergence

Given the clinical overlap between ME/CFS and LC, differential abundance analysis was structured to assess both shared disease-versus-control patterns and differences between the two patient groups. Pairwise contrasts for HC vs. ME/CFS, HC vs. LC, and LC vs. ME/CFS were therefore interpreted as complementary parts of the same three-group design. The raw significant signals are provided in Supplementary Tables S2–S4.

**Figure 3:**
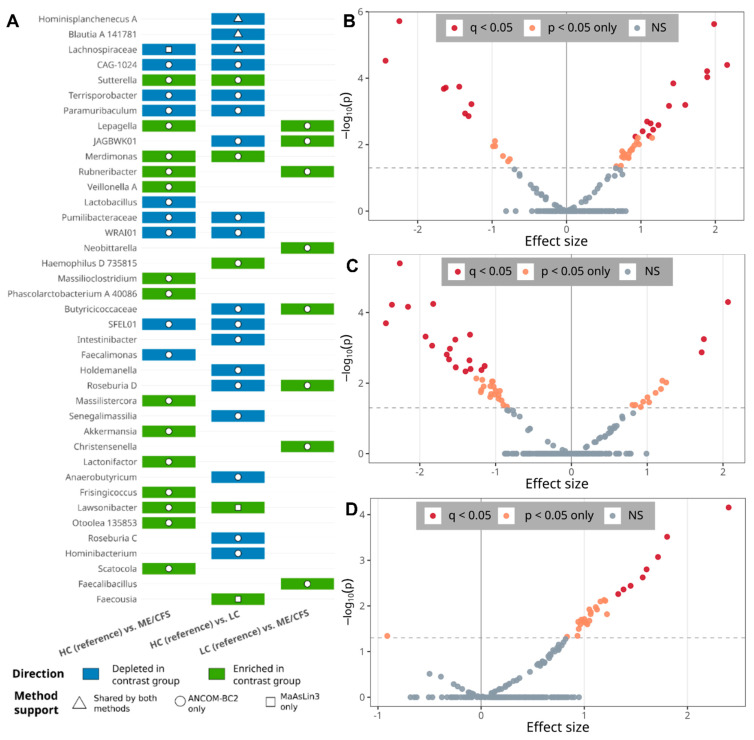
Genus-level DA results from adjusted ANCOM-BC2 and MaAsLin3 on all three contrasts. Only Taxa and labels with q values < 0.05 were displayed. Panel (**A**) shows adjusted genus-level taxa with q < 0.05 detected by ANCOM-BC2, MaAsLin3, or both across the three pairwise contrasts. Triangle orientation indicates whether the taxon is higher in the reference group or the contrast group. Size reflects relative absolute effect magnitude within method, not direct cross-method comparability. Panels (**B**–**D**) show the corresponding adjusted ANCOM-BC2 genus-level volcano plots for each contrast as follows: (**B**) HC (reference) vs. ME/CFS, (**C**) HC (reference) vs. LC, and (**D**) LC (reference) vs. ME/CFS, providing the full effect-size and significance landscape.

**Figure 4:**
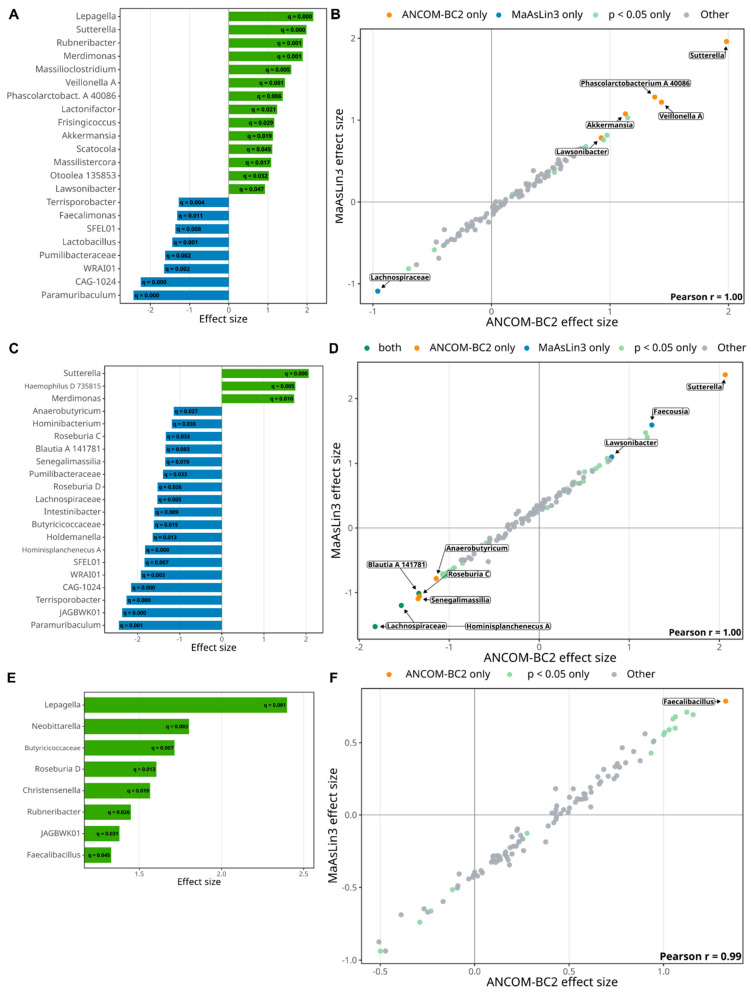
Genus-level agreement between ANCOM-BC2 and MaAsLin3 in pairwise disease contrasts. Panels (**A**,**B**) correspond to HC (reference) vs. ME/CFS, panels (**C**,**D**) to HC (reference) vs. LC, and panels (**E**,**F**) to LC (reference) vs. ME/CFS. Panels (**A**,**C**,**E**) summarize the genera reaching ANCOM-BC2 q < 0.05, with effect direction shown relative to the stated reference group. Panels (**B**,**D**,**F**) compare the corresponding ANCOM-BC2 and MaAsLin3 genus-level effect estimates, highlighting taxa supported by both methods or by either of them. Displayed taxa labels are only from taxa with q value < 0.05. Across contrasts, the scatter plots show high directional concordance between methods, indicated by the Pearson index value in the bottom right corners. ANCOM-BC2 yielded a larger number of significant genus-level findings, particularly in the disease-versus-control comparisons. Because the scatter plots display only matched genera present in both ANCOM-BC2 and MaAsLin3 outputs, taxa identified only by ANCOM-BC2 are not shown when no corresponding MaAsLin3 effect estimate is available.

At the genus level, ANCOM-BC2 identified a broader set of q-significant taxa than MaAsLin3, particularly in the disease-versus-control contrasts (Figure 3A). In the HC reference vs. ME/CFS contrast, ANCOM-BC2 identified enrichment of several genera, including *Sutterella*, *Akkermansia*, *Lawsonibacter*, *Rubneribacter*, *Lepagella*, and *Merdimonas*, whereas *Lactobacillus* and *Paramuribaculum* were among the depleted taxa (Figure 4A). MaAsLin3 retained a smaller signal in the same contrast, with depletion of *Lachnospiraceae* in ME/CFS (Figure 4B).

In the HC reference vs. LC contrast, ANCOM-BC2 again identified multiple genus-level differences. *Sutterella* was enriched in LC, whereas *Terrisporobacter*, *Dorea_A*, *Butyricicoccaceae*, *Anaerobutyricum*, and *Hominisplanchenecus_A* were depleted (Figure 4C). MaAsLin3 identified a more restricted q-significant set, including depletion of *Blautia_A_141781*, *Hominisplanchenecus_A*, and *Lachnospiraceae*, together with enrichment of *Lawsonibacter* and *Faecousia* (Figure 4D).

Across contrasts, ANCOM-BC2 and MaAsLin3 showed strong directional agreement. Matched genus-level effect estimates were highly correlated, with Pearson correlations close to 0.99–1.00 across all three comparisons (Figure 4B,D,F). All data is shown in Supplementary Tables S5 and S6. Thus, the main difference between the methods was the number of taxa retained after multiple-testing correction rather than the direction of the effects. Because ANCOM-BC2 and MaAsLin3 differ in their statistical assumptions and sensitivity to sparsity, compositionality, covariates, and multiple-testing correction, both methods were used as a robustness check, with cross-method agreement interpreted as stronger support.

The shared disease-versus-control component was most evident for four genus-level taxa. *Sutterella* was enriched in both ME/CFS and LC relative to HC, whereas *Terrisporobacter* and *Lachnospiraceae* were depleted in both patient groups. *Lawsonibacter* also showed a shared direction of enrichment, although the strength of statistical support varied by method and contrast. Additional genera showed concordant disease-versus-control direction, including enriched *Merdimonas*, *Veillonella_A*, and *Akkermansia*, and depleted *Paramuribaculum*, *Pumilibacteraceae*, *CAG-1024*, *WRAI01*, *SFEL01*, *Lactobacillus*, *Faecalimonas*, *Intestinibacter*, and *JAGBWK01*. Although these additional taxa were not uniformly q-significant across both disease-versus-control contrasts, their consistent direction supports the broader pattern of shared microbiota traits in the two patient groups.

Species-level analyses were used as supportive information only. ANCOM-BC2 identified several q-significant EMU-assigned species-level features, whereas MaAsLin3 did not identify q-significant species-level hits. For example, the genus-level enrichment of *Akkermansia* in ME/CFS was accompanied by a supportive species-level signal for *Akkermansia muciniphila*. These findings are shown in Supplementary Figures S4 and S5 and were interpreted as exploratory support for the genus-level results.

Overall, the corrected differential abundance analysis supports a distributed genus-level compositional shift rather than a single dominant taxon separating the groups. Disease-versus-control contrasts showed the strongest signal, whereas the direct ME/CFS vs. LC contrast showed fewer differences and weaker cross-method support. This pattern is consistent with partial overlap between ME/CFS and LC gut microbiota profiles, together with selected contrast-specific differences. We added heatmaps of q-significant taxa across samples, showing that the signals were distributed across multiple samples and are not a result of randomness (Supplementary Figure S6).

### 3.5. Integrated Classification and Discrimination Interpretation

**Figure 5:**
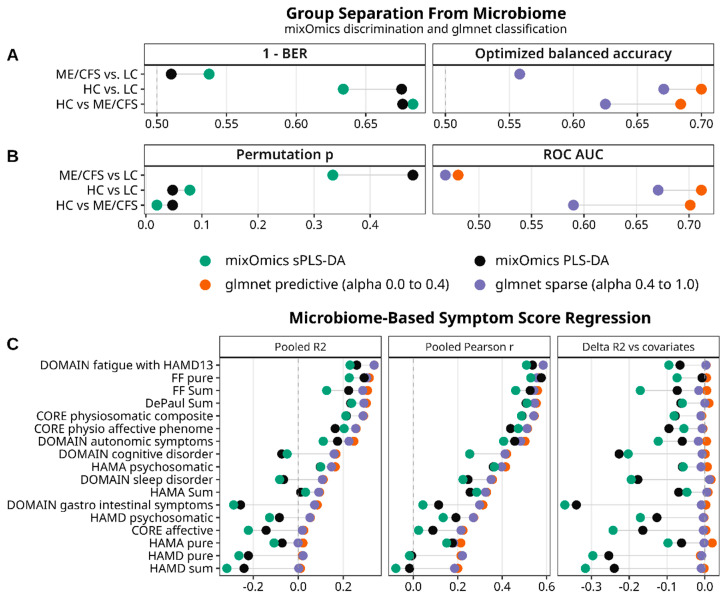
Multivariate microbiome modeling summary. Pairwise group-separation performance from microbiome profiles using mixOmics discrimination (**A**) and glmnet classification (**B**). For mixOmics, separation is shown as 1—BER, so higher values indicate lower balanced error. For glmnet, separation is shown as optimized balanced accuracy. Complementary validation metrics for the same contrasts. mixOmics results are shown with permutation *p* values, whereas glmnet results are shown with ROC AUC. (**C**) Microbiome-based regression of clinical scores using glmnet and mixOmics. Panels show pooled out-of-fold R2, pooled Pearson correlation, and delta R2 relative to the covariates-only model. Positive delta R2 values indicate added explanatory value from the microbiome.

We next focused the multivariate analyses on a screened set of clinically relevant continuous symptom-domain measures, enabling microbiome associations to be examined beyond diagnosis alone. In mixOmics discrimination, HC vs. ME/CFS showed the clearest signal, with sparse sPLS-DA yielding a balanced error rate (BER) of 0.316 and a permutation *p* value of 0.020, while dense PLS-DA gave a BER of 0.324 with a permutation *p* value of 0.048 (Figure 5A,B). HC vs. LC was also moderately separable, although support was less uniform across methods. Dense PLS-DA gave a BER of 0.324 with permutation *p* = 0.048, whereas sparse sPLS-DA gave a BER of 0.366 with permutation *p* = 0.078. By contrast, ME/CFS vs. LC was weak in both sparse and dense discrimination models (BER 0.463–0.490; permutation *p* = 0.333–0.476), indicating little robust multivariate separation after adjustment for age, BMI, and sex.

Adjusted glmnet classification supported the same overall pattern. In the predictive ridge branch, pooled out-of-fold ROC AUC was 0.701 for HC vs. ME/CFS, 0.712 for HC vs. LC, and only 0.480 for ME/CFS vs. LC; the corresponding optimized balanced accuracies were 0.684, 0.700, and 0.558, respectively. The sparse branch was weaker and did not retain any microbiome features under the conservative lambda.1se rule for any pairwise contrast, with only exploratory lambda.min solutions for the two disease-versus-control contrasts (five taxa for HC vs. ME/CFS and 10 for HC vs. LC) and no retained features for ME/CFS vs. LC. Taken together, discrimination and classification agreed on the same conclusion that disease-versus-control pairs are only modestly separable, whereas direct ME/CFS-versus-post-COVID separation is weak. The stronger performance of dense over sparse models also argues that the signal is broad and distributed across many taxa rather than driven by a small stable microbial signature. Detailed results are provided in Supplementary Table S7.

### 3.6. Clinical Phenotype Regression

Across adjusted regression models, the strongest microbiome-associated outcomes were fatigue- and physiosomatic-related composites rather than affective or gastrointestinal domains. In glmnet predictive regression, the highest pooled out-of-fold R^2^ values were observed for DOMAIN-fatigue-with-HAMD13 (0.338), DePaul-Sum (0.303), CORE-physiosomatic-composite (0.290), CORE-physio-affective-phenome (0.257), and DOMAIN-autonomic-symptoms (0.247), with corresponding Pearson correlations around 0.50–0.58 (Figure 5C). mixOmics regression showed a similar rank order but lower absolute performance, with top pooled R^2^ values in the range of approximately 0.21–0.26 for fatigue, DePaul-Sum, physiosomatic, and related composite outcomes in the dense and sparse PLS frameworks. This suggests that microbiome variation tracks some symptom dimensions more than others, particularly fatigue-related and physiosomatic burden. Exact data is shown in Supplementary Table S8.

However, the key result is that microbiome-added value beyond covariates was small and method-dependent. In glmnet regression, delta R^2^ over the covariate-only baseline was positive for some outcomes, but the gains were modest, around 0.00–0.02. In contrast, mixOmics regression did not provide incremental benefit beyond covariates. In the sparse sPLS branch, delta R^2^ was negative across all outcomes, and the dense PLS branch showed the same overall pattern. Several domains, particularly affective, gastrointestinal, and HAMD-related measures, had near-zero or negative pooled out-of-fold R^2^ in mixOmics, arguing against a stable microbiome contribution after adjustment for age, BMI, sex, and patient group. Overall, the regression analyses support limited microbiome-symptom coupling, strongest for fatigue and physiosomatic domains, but supportive of strong independent predictive utility.

### 3.7. Selected Predicted Functional Pathways

**Figure 6:**
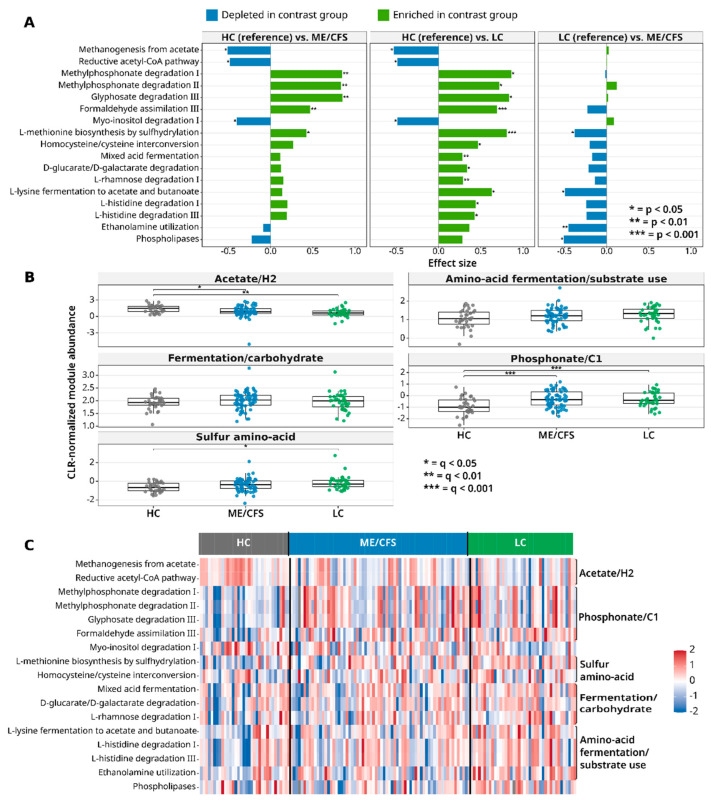
Selected PICRUSt2-predicted functional pathway differences across HC, ME/CFS, and LC. (**A**) MaAsLin3 effect plots for selected predicted pathways across pairwise contrasts. Positive values (green) indicate enrichment in the contrast group relative to the reference group, whereas negative values (blue) indicate depletion in the contrast group. (**B**) Module-level predicted pathway abundances, generated by summing selected pathways within each module and testing them with adjusted MaAsLin3 models; significance reflects Benjamini–Hochberg FDR-corrected pairwise contrasts. (**C**) Heatmap of row-z-scored CLR-transformed selected PICRUSt2-predicted pathway abundances across samples, shown to illustrate sample-level distribution patterns across groups.

To test whether taxonomic differences would lead to a change in the functional potential of the microbiome, we added an exploratory functional layer based on PICRUSt2, which predicts the abundance of metabolic pathways (i.e., the predicted functional potential). From the full predicted pathway profile, 261 pathways were submitted to MaAslin3 after prevalence filtering. As a result, between 16 and 66 pathways between the three contrasts were rendered nominally significant (*p* < 0.05). Of these, we displayed 17 in Figure 6. Housekeeping and core biosynthetic pathways (e.g., tRNA charging, nucleotide and amino acid biosynthesis, glycolysis, etc.) were omitted.

Individual pathway-level contrasts from MaAslin3 (HC vs. ME/CFS, HC vs. LC, and LC vs. ME/CFS) are displayed in Figure 6A. Both disease groups showed a broadly overlapping disease-versus-control pathways pattern and directionality. However, the profiles were not identical, with LC showing broader nominal enrichment of several fermentation- and substrate-use-related pathways such as mixed acid fermentation, D-glucarate/D-galactarate degradation, L-rhamnose degradation, L-lysine fermentation to acetate and butanoate, and histidine degradation pathways. Both ME/CFS and LC had lower predicted abundance of acetate/H_2_ carbon flow pathways (e.g., methanogenesis from acetate, reductive acetyl-CoA pathway) and myo-inositol degradation compared to HC. The direct ME/CFS–LC contrast showed more limited disease-specific differences. Some amino acid fermentation/mucosal-substrate and lipid-related pathways were lower in ME/CFS (e.g., lysine and histidine fermentation, phospholipase pathway).

As a next step, we grouped some of the selected PICRUSt2-predicted pathways into predefined functional modules to reduce reliance on isolated nominal pathway-level signals and redundancy and to test whether related inferred pathway differences translate into broader gut-relevant categories. After summing raw pathway abundances per module and rerunning via MaAsLin3, the main disease-versus-control pattern became clearer. Both ME/CFS and LC showed lower acetate/H_2_ carbon flow and higher phosphonate/C1-related predicted metabolism modules relative to HC. Importantly, these module-level differences remained significant after BH-FDR correction. In contrast, none of the direct ME/CFS vs. LC module comparisons remained significant after correction, suggesting that the strongest predicted functional signal was shared by both disease groups relative to HC rather than clearly separating ME/CFS from LC. The heatmap in Figure 6C illustrates that selected pathway signals occur across multiple samples.

Overall, the PICRUSt2 layer supports overlapping disease-vs-control remodeling: ME/CFS and LC both show lower acetate/H_2_ and inositol/polyol potential and higher phosphonate/C1-related potential relative to HC, with LC showing nominally broader enrichments. These predicted functional potential patterns are consistent with shared gut-microbial shifts but should be viewed as exploratory and inferred from predictions.

## 4. Discussion

The clinical similarity of ME/CFS and LC remains a challenge for modern medicine, requiring an examination of their shared biological basis. The strength of this study is the simultaneous analysis of both cohorts within a single framework, as these post-infectious states are often studied separately but they share common symptom traits. This work is strengthened by the use of a relatively large clinical cohort for Bulgaria and by the direct comparison of fecal microbiome signatures across both conditions [3]. It provides a basis for future microbiome-informed stratification, suggesting that although the initial triggers differ, both conditions may converge on related dysbiotic patterns. The focus on overlap is important for future interventions, as evidence suggests these conditions are related but non-identical in their microbial composition [9,44].

In the present study, ME/CFS and LC showed a broadly shared baseline microbial composition, with only modest changes in individual taxa. Some known anaerobic commensals, including members of *Lachnospiraceae* such as *Blautia*, were lower in the disease groups relative to controls, whereas other taxa, including *Akkermansia* and *Veillonella*, were increased in ME/CFS in compared to the HC. These observations are broadly consistent with previous reports that ME/CFS is often associated with reduced *Firmicutes* relative to *Bacteroidetes* [39], while long COVID has also been linked to depletion of “beneficial” butyrate producers and enrichment of proinflammatory genera such as *Veillonella* [41]. Moreover, *Sutterella* and *S. wadsworthensis* were also enriched in similar studies in COVID patients and were associated with persistent symptoms such as six-month fatigue [45]. In both our disease groups *Sutterella* and *S. wadsworthensis* were enriched consistently, directly aligning to other study findings. The regression branch of the multivariate analysis showed that fatigue- and physiosomatic-related outcomes were the symptom domains most strongly associated with gut microbiome variation. *Sutterella* is known for degrading IgA [46], with potential relevance to the intestinal immune barrier [10]. However, no particular evidence currently exists to support its role toward disrupting epithelial homeostasis [47].

*Lachnospiraceae*, another significantly depleted genus in both our disease cohorts was described as having a controversial role in human gut with either found enriched or depleted in various conditions including inflammation related states [48]. It is also positively associated with vegetable diets and negatively associated with omnivore diet [49]. Therefore, without complete knowledge on diet or dietary habits of the current cohorts, the interpretation remains limited. However, it should be noted that previous studies in COVID-19 have described a severity-related decline in *Lachnospiraceae* and *Roseburia*, suggesting that depletion of these anaerobic commensals becomes more pronounced with increasing severity of acute SARS-CoV-2 infection. Other authors have found that the severity of the acute COVID-19 was the strongest predictor of LC risk, therefore *Lachnospiraceae* was proposed as biomarker candidate for assessing severity and potentially for LC predictor [50].

*Terrisporobacter* was significantly depleted in both diseases. However, this genus should not be interpreted as beneficial or harmful. Published findings on *Terrisporobacter* appear context-dependent, with reports linking it to both protective [51] and disease-promoting [52] settings in different conditions. Therefore, its depletion in our cohorts is best viewed as a shared disease-associated compositional marker rather than direct evidence of functional loss.

We attempted to look for differences or shared traits in the functions of the microbiome of sample cohorts. However, this functional analysis should be interpreted primarily as predicted rather than directly measured activity or metabolite concentrations. Among the selected pathways, the anaerobic acetate/H_2_-carbon-flow signal is particularly relevant to gut microbial fermentation, because the reductive acetyl-CoA/Wood–Ljungdahl pathway contributes to acetate production and hydrogen disposal in intestinal microbial communities [53]. Acetate is a central SCFA and substrate in the gut, while hydrogen disposal helps maintain efficient anaerobic fermentation. Therefore, changes in this pathway may indicate altered fermentation balance and microbial cross-feeding capacity. The anaerobic acetate/H_2_-carbon-flow module was significantly reduced in both ME/CFS and LC relative to HC, suggesting that both disease groups shared a lower predicted capacity for anaerobic acetate-linked carbon flow and hydrogen disposal.

Inositol/polyol degradation was another shared disease-vs-control signal. This pathway is relevant because gut bacteria can use dietary or host-associated inositol-derived compounds as carbon and energy sources, and intestinal bacteria have been shown to convert inositol isomers into SCFA such as propionate and acetate [54,55]. In our results, this predicted pathway was depleted in both ME/CFS and LC relative to HC, suggesting reduced relative predicted capacity for inositol/polyol substrate utilization in both disease groups. This may reflect loss or lower relative abundance of bacteria carrying this metabolic trait. Inositol-derived compounds are common in plant-derived foods, especially seeds, grains, legumes, nuts, and bran-rich cereals [56]. Its significant depletion in both diseases aligns with the lower levels of *Lachnospiraceae* and could be both caused by dietary differences across our cohorts.

Phosphonate/C1-related predicted metabolism was one of the strongest shared module-level signals. Briefly, phosphonate-degradation pathways allow bacteria to break stable C–P bonds and use organophosphonates as phosphorus sources, while formaldehyde-related C1 pathways help bacteria assimilate or detoxify reactive one-carbon intermediates such as formaldehyde [57]. In our results, this module was enriched in both ME/CFS relative to HC and LC relative to HC, with FDR-supported module-level effects in both contrasts, indicating a shared disease-vs-control predicted functional pattern. This could reflect enrichment of bacteria with broader nutrient-scavenging/C1-stress-handling capacity, or a relative increase due to other community members decreasing.

L-lysine fermentation to acetate and butyrate is also relevant because it represents an amino-acid-derived route for microbial SCFA production, complementing carbohydrate-derived fermentation pathways [58]. Human colonic butyrate production can arise from both carbohydrate and amino acid fermentation, and lysine is among recognized bacterial routes to butyrate synthesis [59]. In our results, this pathway was enriched in LC relative to HC and depleted in ME/CFS relative to LC, suggesting higher predicted lysine-derived acetate/butyrate fermentation potential in LC. Because the broader amino acid fermentation/substrate-use module was not FDR-significant, this signal remains exploratory. Guo et al. also showed deficiencies in lysine pathway in patients with ME/CFS [10].

In the regression analyses, fatigue-related and physiosomatic domains showed the strongest association with genus-level microbiome profiles, whereas affective, cognitive, and gastrointestinal outcomes showed weaker or less stable signals. This pattern is consistent with previous ME/CFS microbiome studies in which microbial alterations, especially reduced butyrate-producing capacity and altered bacterial network structure, were linked to fatigue symptoms rather than only to diagnostic status [10]. Similarly, Xiong et al. reported gut microbiome differences in ME/CFS together with changes in host metabolic and immune features, supporting the view that microbiome alterations are part of a wider biological disturbance rather than an isolated finding [11]. In LC, persistent symptoms have similarly been associated with altered gut microbiota composition, reduced microbial diversity, and poorer patient-reported quality of life and higher anxiety/depression scores in longitudinal and post-discharge cohorts [21,27]. Our findings therefore fit with the emerging view that microbiome variation may track fatigue and overall physical illness burden in post-infectious syndromes. However, the microbiome-added explanatory value beyond covariates was small and method-dependent, indicating that these associations should not be interpreted as a fatigue biomarker.

In essence, the main conclusion is that ME/CFS and LC represent related, broadly overlapping but not identical microbiome-associated states. These results are compatible with models linking dysbiosis to physio-affective symptoms, although not linking such pathways directly. Future research should transition to longitudinal designs using shotgun metagenomics to resolve the species-level nuances identified. Integration with multi-omics data, such as fecal metabolomics for SCFA concentrations, is needed to confirm links between barrier breakdown and symptoms. Stratification strategies based on disease duration will be important for validating the stability of these microbial configurations. While microbiome-based diagnostics remain exploratory, these findings may help inform future validation studies and therapeutic hypothesis generation.

Several limitations of this study should be acknowledged. First, the cross-sectional design prevents the determination of causality regarding whether microbial shifts precede or follow symptoms. Second, the lack of longitudinal monitoring means the study could not capture temporal instability in post-infectious syndromes. Third, although recent antibiotic, corticosteroid, immunosuppressive, and immune-modulating medication use were exclusion criteria, detailed information on other key microbiome-modifying factors such as habitual diet, probiotic or prebiotic use, nutritional supplements, other medications, alcohol consumption, and smoking was incomplete. As a result, residual confounding cannot be excluded. Fourth, severely affected, bedbound ME/CFS patients were underrepresented due to the voluntary nature of clinic attendance. Fifth, partial missingness in clinical metadata and questionnaire completion reduced the power for association analyses.

## 5. Conclusions

In this study, ME/CFS and LC were compared with HC within a single analytical framework combining. ME/CFS and LC showed overlapping but non-identical gut microbiota patterns. The strongest signals were observed in disease-versus-control comparisons, while the direct ME/CFS vs. LC contrast showed fewer differences. At the genus level, both disease groups shared enrichment of *Sutterella* and *Lawsonibacter* and depletion of *Terrisporobacter* and *Lachnospiraceae*. Species-level results supported selected genus-level findings, and predicted functional profiling showed shared changes in pathways related to anaerobic acetate/H_2_ carbon flow, inositol/polyol degradation, phosphonate/C1-related metabolism, and lysine-derived fermentation. Regression analyses showed that microbiome profiles were most strongly associated with fatigue-related and physiosomatic domains, whereas affective, cognitive, and gastrointestinal outcomes showed weaker signals. Overall, these findings indicate that ME/CFS and LC share part of their gut microbiota disturbance. Future studies using larger longitudinal cohorts, improved control of diet and medication exposure, shotgun metagenomics, metabolomics, and immune or gut barrier markers are needed to validate and clarify the biological relevance of these patterns.

## Figures and Tables

**Table 1:** Demographic and anthropometric characteristics of the study cohort.

Variable	HC	ME/CFS	LC	Overall	Raw *p* Value	FDR	Significant Pairwise
N	37	76	50	163			
Female, n (%)	26 (70.3%)	59 (77.6%)	37 (74.0%)	122 (74.8%)	0.689	0.862	
Male, n (%)	11 (29.7%)	17 (22.4%)	13 (26.0%)	41 (25.2%)			
Age, median [IQR], n	33.00 [23.00–47.00], n = 37	45.00 [33.00–54.50], n = 76	46.50 [39.00–57.00], n = 50	44.00 [32.00–55.00], n = 163	0.001	0.003	HC vs. ME/CFS (FDR = 0.005); HC vs. LC (FDR = 0.001)
Weight, median [IQR], n	62.50 [54.00–80.00], n = 34	64.50 [58.50–77.50], n = 68	67.50 [58.50–81.50], n = 40	65.00 [58.00–80.00], n = 142	0.379	0.631	
Height, median [IQR], n	165.00 [163.00–175.00], n = 34	168.50 [161.50–176.00], n = 68	167.00 [164.00–172.00], n = 40	167.00 [162.00–175.00], n = 142	0.981	0.981	
BMI, median [IQR], n	22.16 [19.15–27.17], n = 34	23.44 [20.96–25.91], n = 68	23.98 [21.89–27.83], n = 40	23.48 [20.58–27.10], n = 142	0.368	0.631	

IQR—interquartile range. Continuous variables are shown as median [IQR] with available N; categorical variables are shown as n (%). Group comparisons used Kruskal–Wallis tests for continuous variables and χ^2^ or Fisher’s exact tests for categorical variables, with Benjamini–Hochberg FDR correction across baseline variables. For variables with missing data, available N is shown in the table. Detailed sample-level metadata and BMI-category summaries are provided in Supplementary Table S1.

**Table 2:** Beta-diversity analyses across the full cohort and the complete metadata subset.

Analysis Set	Distance	Test/Model	Covariates	N	Statistic	R^2^	*p* Value	Note
Full cohort	Bray–Curtis	PERMANOVA	none	163	pseudo-F = 1.499		0.017	Global group difference
Full cohort	Bray–Curtis	PERMDISP	none	163	F = 1.402	–	0.270	No significant dispersion difference
Full cohort	Jaccard	PERMANOVA	none	163	pseudo-F = 1.326		0.023	Presence/absence-based group difference
Complete metadata subset	Bray–Curtis	adonis2	patient_group	142	F.Model = 1.644	0.0231	0.003	Unadjusted disease effect
Complete metadata subset	Bray–Curtis	adonis2	age + BMI + sex + patient_group	142	F.Model = 1.858	0.0260	0.001	Disease effect remains significant after adjustment

N indicates the number of post-QC samples retained in each analysis; covariate-adjusted adonis2 models were restricted to samples with complete age, BMI, and sex metadata. Bray–Curtis distance summarizes abundance-weighted compositional dissimilarity, whereas Jaccard distance summarizes presence/absence-based taxon turnover. PERMANOVA tests whether group centroids differ in multivariate community space, while PERMDISP tests whether groups differ in within-group dispersion. R^2^ indicates the proportion of community-level variation explained by the tested variable.

## Data Availability

All raw 16S rRNA amplicon sequence data derived from this study have been submitted to NCBI BioProject under Accession PRJNA1438146. The other human datasets used are available from the corresponding author on request after IRB approval.

## Supplementary Materials

The following supporting information can be downloaded at: https://www.mdpi.com/article/10.3390/biomedicines14061183/s1, Supplementary files description; Supplementary Figure S1: Sequencing depth and rarefaction overview; Supplementary Figure S2: Phylum-level taxonomic composition; Supplementary Figure S3: Descriptive unsupervised ordination plots; Supplementary Figure S4: Species-level differential abundance results; Supplementary Figure S5: Species-level agreement between ANCOM-BC2 and MaAsLin3; Supplementary Figure S6: Sample-level heatmaps of ANCOM-BC2; Table S1: Patient metadata, Table S2: ANCOM_genus_adj_sig, Table S3: ANCOM_species_adj_sig, Table S4: MaAsLin_genus_adj_sig, Table S5: Consensus_genus_adj, Table S6: Consensus_species_adj, Table S7: Multivar_group_sep, Table S8: Multivar_regression.
