# Supplementary material for: Comparative Gut Microbiome Alterations in Myalgic Encephalomyelitis/Chronic Fatigue Syndrome and Long COVID-19 Syndrome
Source: Biomedicines. 2026 May 22;14(6):1183. doi: 10.3390/biomedicines14061183 (PMC13296197; doi:10.3390/biomedicines14061183)
Supplement: Supplementary file 1 [file biomedicines-14-01183-s001.zip › suppl. files/Supplementary Figure S3. Descriptive unsupervised ordination plots.pdf]

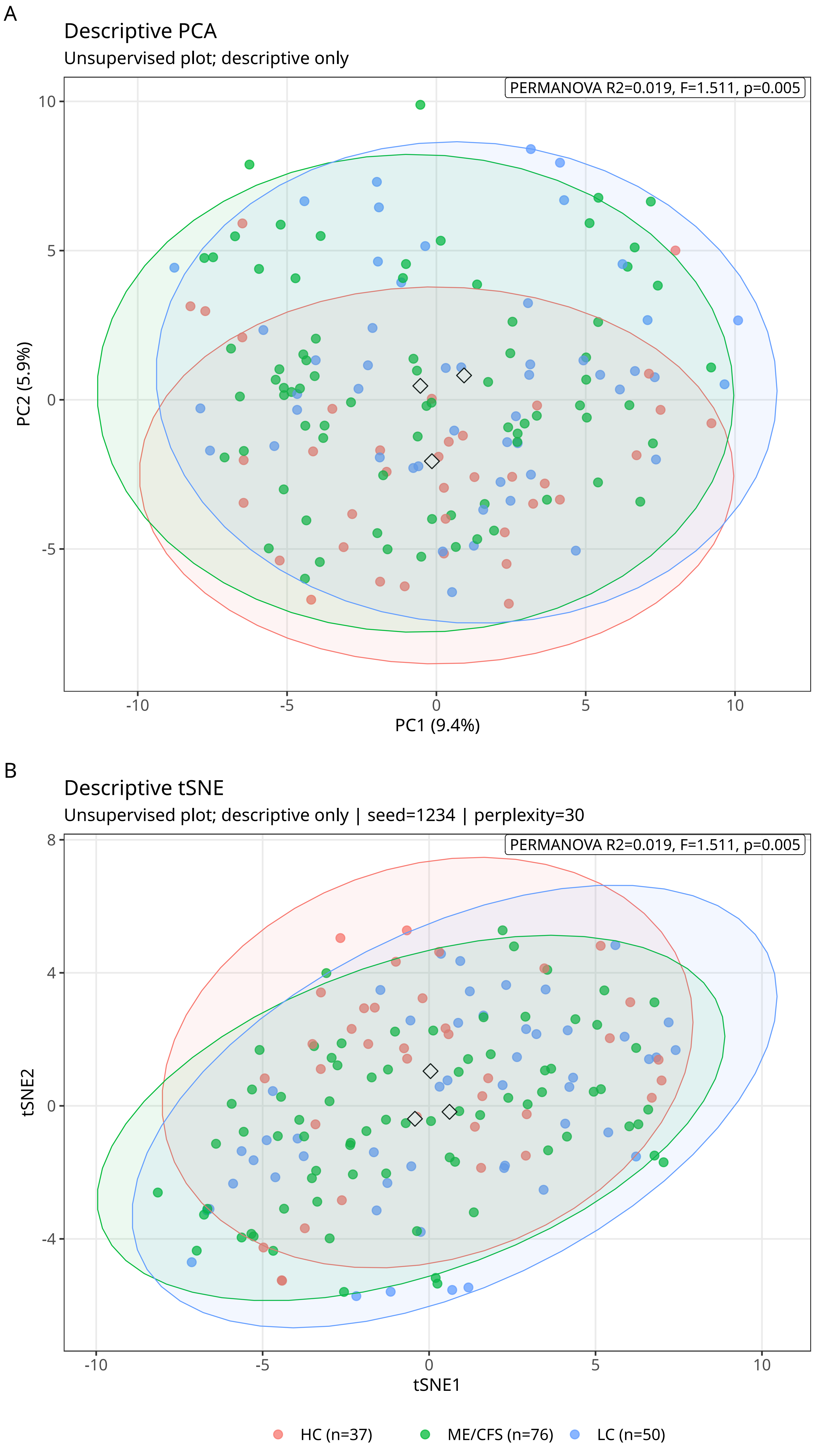
A
Descriptive PCA
Unsupervised plot; descriptive only
PERMANOVA R2=0.019, F=1.511, p=0.005
PC2 (5.9%)
PC1 (9.4%)
10
5
0
-5
-10
-5
0
5
10
B
Descriptive tSNE
Unsupervised plot; descriptive only | seed=1234 | perplexity=30
PERMANOVA R2=0.019, F=1.511, p=0.005
tSNE2
tSNE1
8
4
0
-4
-10
-5
0
5
10
HC (n=37)
ME/CFS (n=76)
LC (n=50)
