# Supplementary material for: Comparative Gut Microbiome Alterations in Myalgic Encephalomyelitis/Chronic Fatigue Syndrome and Long COVID-19 Syndrome
Source: Biomedicines. 2026 May 22;14(6):1183. doi: 10.3390/biomedicines14061183 (PMC13296197; doi:10.3390/biomedicines14061183)
Supplement: Supplementary file 1 [file biomedicines-14-01183-s001.zip › suppl. files/Supplementary files description.docx]

**Supplementary Figure S1.** Sequencing depth and rarefaction overview.
Panel **A** shows the distribution of read counts per sample after processing. Panel **B** shows alpha-rarefaction curves based on observed features, indicating that most samples approached a plateau before the selected rarefaction depth.

**Supplementary Figure S2.** Phylum-level taxonomic composition.
Stacked barplots show the relative abundance of dominant phylum-level taxa across samples, grouped by cohort. The plot provides a broad compositional overview and supports the absence of a gross disease-specific phylum-level shift.

**Supplementary Figure S3.** Descriptive unsupervised ordination plots.
PCA and t-SNE plots show broad overlap among HC, ME/CFS, and LC samples, consistent with subtle community-level differences rather than discrete group separation. PERMANOVA results are shown on the plots for reference.

**Supplementary Figure S4.** Species-level differential abundance results from adjusted ANCOM-BC2 and MaAsLin3 on all three contrasts. Only Taxa and labels with q values < 0.05 were displayed. **A.** shows adjusted genus-level taxa with q < 0.05 detected by ANCOM-BC2, MaAsLin3, or both across the three pairwise contrasts. Triangle orientation indicates whether the taxon is higher in the reference group or the contrast group. Size reflects relative absolute effect magnitude within method, not direct cross-method comparability. Panels **B–D** show the corresponding adjusted ANCOM-BC2 genus-level volcano plots for each contrast as follows **B.** HC (reference) vs. ME/CFS, **C.** HC (reference) vs. LC, and **D.** LC (reference) vs. ME/CFS, providing the full effect-size and significance landscape.

**Supplementary Figure S5.** Species-level agreement between ANCOM-BC2 and MaAsLin3 in pairwise disease contrasts. Panels **A.** and **B.** correspond to HC (reference) vs. ME/CFS, panels **C.** and **D.** to HC (reference) vs. LC, and panels **E.** and **F.** to LC (reference) vs. ME/CFS. Panels A, C, and E summarize the genera reaching ANCOM-BC2 q < 0.05, with effect direction shown relative to the stated reference group. Panels B, D, and F compare the corresponding ANCOM-BC2 and MaAsLin3 genus-level effect estimates, highlighting taxa supported by both methods or by either of them. Displayed taxa labels are only from taxa with q value < 0.05. Across contrasts, the scatter plots show high directional concordance between methods, indicated by the Pearson index value in the bottom right corners. ANCOM-BC2 yielded a larger number of significant genus-level findings, particularly in the disease-versus-control comparisons. Because the scatter plots display only matched species present in both ANCOM-BC2 and MaAsLin3 outputs, taxa identified only by ANCOM-BC2 are not shown when no corresponding MaAsLin3 effect estimate is available.

## **Supplementary Figure S6.** Sample-level heatmaps of ANCOM-BC2 differentially abundant taxa. Heatmaps show the distribution of ANCOM-BC2 differentially abundant taxa across individual samples. Panels **A–C** show genus-level results for **HC vs ME/CFS**, **HC vs LC**, and **LC vs ME/CFS**, respectively; panels **D–F** show the corresponding species-level results for the same contrasts. Within each panel, taxa enriched in the contrast group are shown in the upper part and taxa depleted in the contrast group in the lower part. Color intensity represents relative abundance, with panel-specific scaling to make low-abundance occurrences visible; therefore, color intensity should not be directly compared across panels. These heatmaps are descriptive sample-level visualizations showing that the main ANCOM-BC2 signals are distributed across multiple individuals rather than driven by a single extreme sample.

**Supplementary Tables contains**: Table S1. Patient metadata, Tables S2 through S7 as follows: S2. ANCOM_genus_adj_sig, S3. ANCOM_species_adj_sig, S4. MaAsLin_genus_adj_sig, S5. Consensus_genus_adj, S6. Consensus_species_adj, S7. Multivar_group_sep, S8. Multivar_regression.
