# Supplementary material for: Comparative Gut Microbiome Alterations in Myalgic Encephalomyelitis/Chronic Fatigue Syndrome and Long COVID-19 Syndrome
Source: Biomedicines. 2026 May 22;14(6):1183. doi: 10.3390/biomedicines14061183 (PMC13296197; doi:10.3390/biomedicines14061183)
Supplement: Supplementary file 1 [file biomedicines-14-01183-s001.zip › suppl. files/Supplementary Figure S6. Sample-level heatmaps of ANCOM-BC2.pdf]

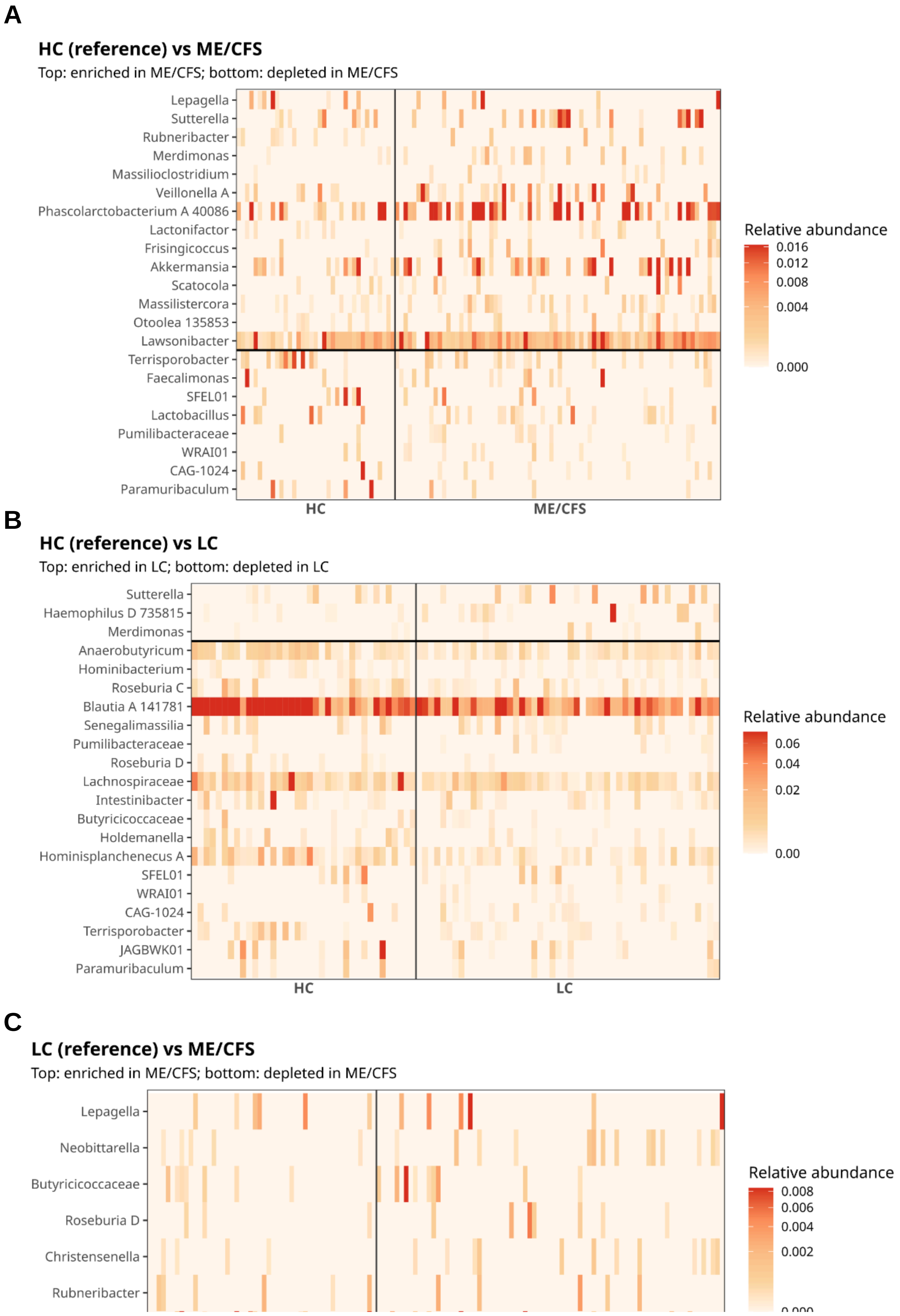
A
HC (reference) vs ME/CFS
Top: enriched in ME/CFS; bottom: depleted in ME/CFS
Lepagella
Sutterella
Rubneribacter
Merdimonas
Massilioclostridium
Veillonella A
Phascolarctobacterium A 40086
Lactonifactor
Frisingicoccus
Akkermansia
Scatocola
Massilistercora
Otoolea 135853
Lawsonibacter
Terrisporobacter
Faecalimonas
SFEL01
Lactobacillus
Pumilibacteraceae
WRAI01
CAG-1024
Paramuribaculum
HC
ME/CFS
Relative abundance
0.016
0.012
0.008
0.004
0.000
B
HC (reference) vs LC
Top: enriched in LC; bottom: depleted in LC
Sutterella
Haemophilus D 735815
Merdimonas
Anaerobutyricum
Hominibacterium
Roseburia C
Blautia A 141781
Senegalimassilia
Pumilibacteraceae
Roseburia D
Lachnospiraceae
Intestinibacter
Butyricicoccaceae
Holdemanella
Hominisplanchenecus A
SFEL01
WRAI01
CAG-1024
Terrisporobacter
JAGBWK01
Paramuribaculum
HC
LC
Relative abundance
0.06
0.04
0.02
0.00
C
LC (reference) vs ME/CFS
Top: enriched in ME/CFS; bottom: depleted in ME/CFS
Lepagella
Neobittarella
Butyricicoccaceae
Roseburia D
Christensenella
Rubneribacter
Relative abundance
0.008
0.006
0.004
0.002

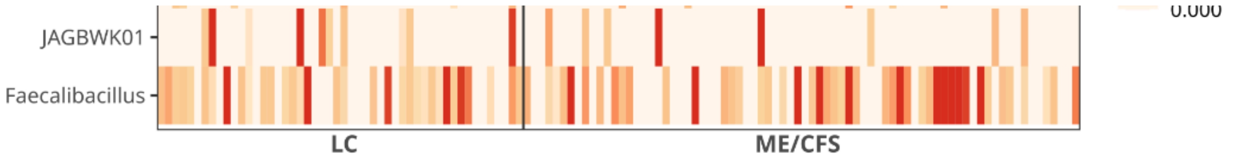
0.000
JAGBWK01
Faecalibacillus
LC
ME/CFS

D

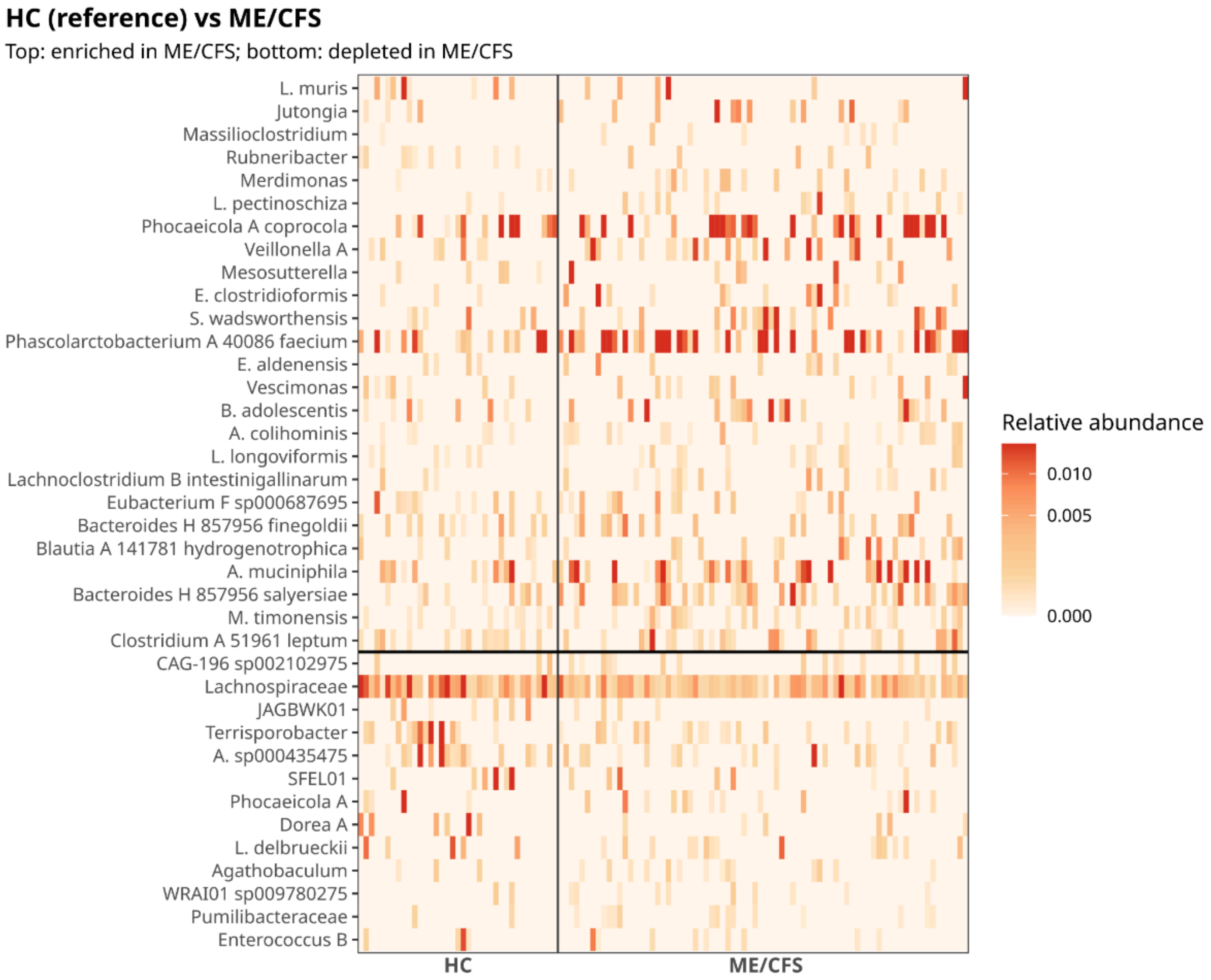
HC (reference) vs ME/CFS
Top: enriched in ME/CFS; bottom: depleted in ME/CFS
L. muris
Jutongia
Massilioclostridium
Rubneribacter
Merdimonas
L. pectinoschiza
Phocaeicola A coprocola
Veillonella A
Mesosutterella
E. clostridioformis
S. wadsworthensis
Phascolarctobacterium A 40086 faecium
E. aldenensis
Vescimonas
B. adolescentis
A. colihominis
L. longoviformis
Lachnoclostridium B intestinigallinarum
Eubacterium F sp000687695
Bacteroides H 857956 finegoldii
Blautia A 141781 hydrogenotrophica
A. muciniphila
Bacteroides H 857956 salyersiae
M. timonensis
Clostridium A 51961 leptum
CAG-196 sp002102975
Lachnospiraceae
JAGBWK01
Terrisporobacter
A. sp000435475
SFEL01
Phocaeicola A
Dorea A
L. delbrueckii
Agathobaculum
WRAI01 sp009780275
Pumilibacteraceae
Enterococcus B
HC
ME/CFS
Relative abundance
0.010
0.005
0.000

E

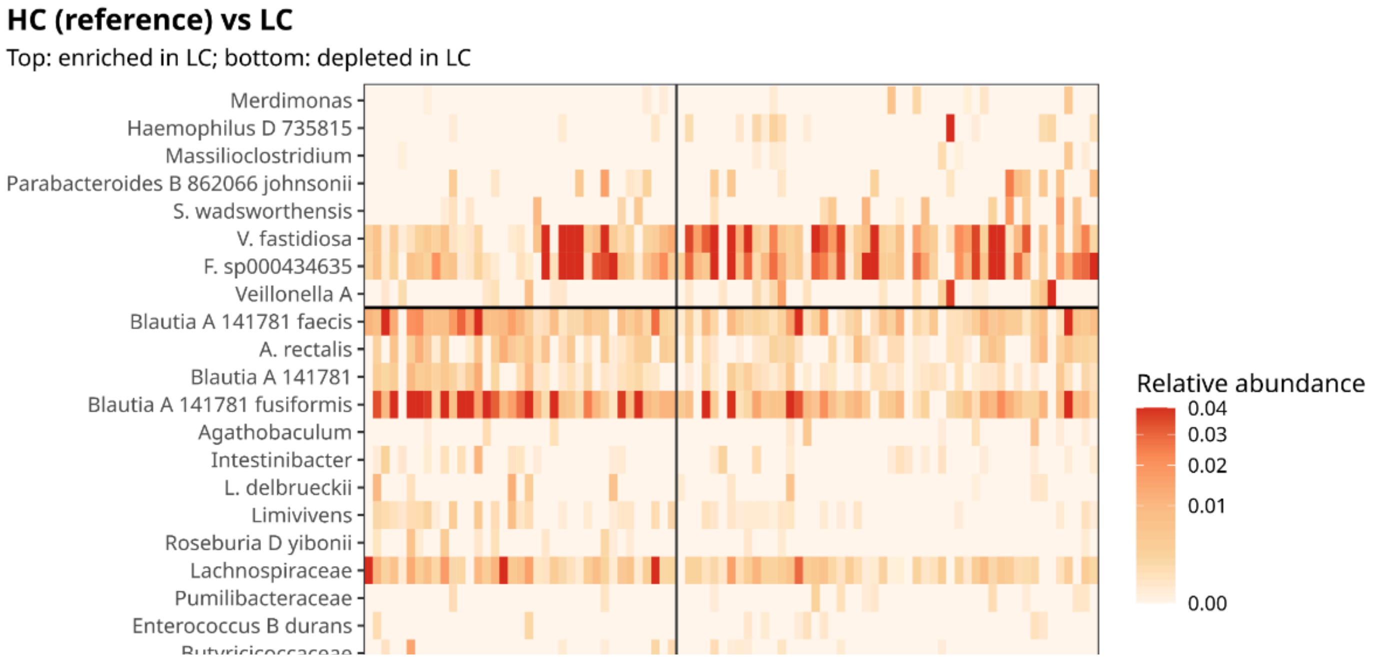
HC (reference) vs LC
Top: enriched in LC; bottom: depleted in LC
Merdimonas
Haemophilus D 735815
Massilioclostridium
Parabacteroides B 862066 johnsonii
S. wadsworthensis
V. fastidiosa
F. sp000434635
Veillonella A
Blautia A 141781 faecis
A. rectalis
Blautia A 141781
Blautia A 141781 fusiformis
Agathobaculum
Intestinibacter
L. delbrueckii
Limivivens
Roseburia D yibonii
Lachnospiraceae
Pumilibacteraceae
Enterococcus B durans
Relative abundance
0.04
0.03
0.02
0.01
0.00

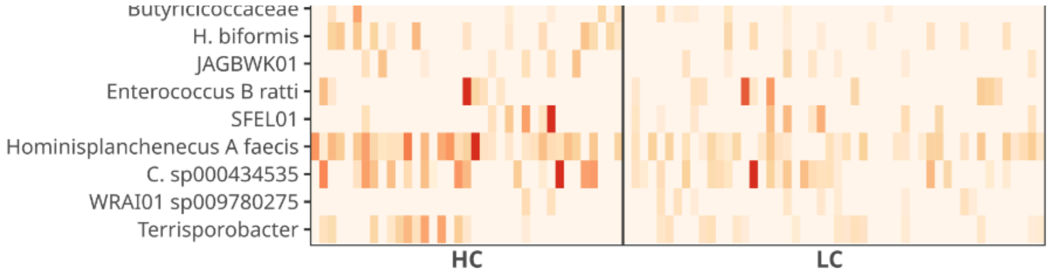
Butyricicoccaceae
H. biformis
JAGBWK01
Enterococcus B ratti
SFEL01
Hominisplanchenecus A faecis
C. sp000434535
WRAI01 sp009780275
Terrisporobacter
HC
LC

F

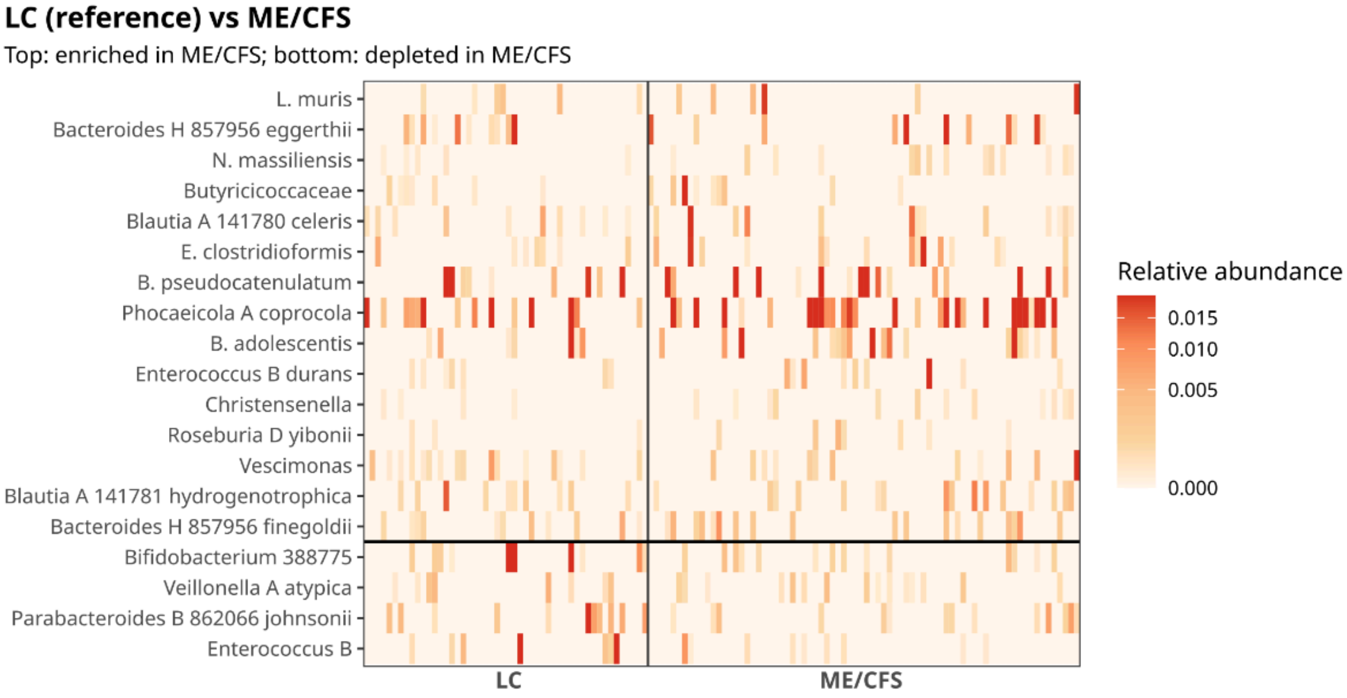
LC (reference) vs ME/CFS
Top: enriched in ME/CFS; bottom: depleted in ME/CFS
L. muris
Bacteroides H 857956 eggerthii
N. massiliensis
Butyricicoccaceae
Blautia A 141780 celeris
E. clostridioformis
B. pseudocatenulatum
Phocaeicola A coprocola
B. adolescentis
Enterococcus B durans
Christensenella
Roseburia D yibonii
Vescimonas
Blautia A 141781 hydrogenotrophica
Bacteroides H 857956 finegoldii
Bifidobacterium 388775
Veillonella A atypica
Parabacteroides B 862066 johnsonii
Enterococcus B
LC
ME/CFS
Relative abundance
0.015
0.010
0.005
0.000
