# Supplementary figures and images for: Comparative Gut Microbiome Alterations in Myalgic Encephalomyelitis/Chronic Fatigue Syndrome and Long COVID-19 Syndrome
Source: Biomedicines. 2026 May 22;14(6):1183. doi: 10.3390/biomedicines14061183 (PMC13296197; doi:10.3390/biomedicines14061183)

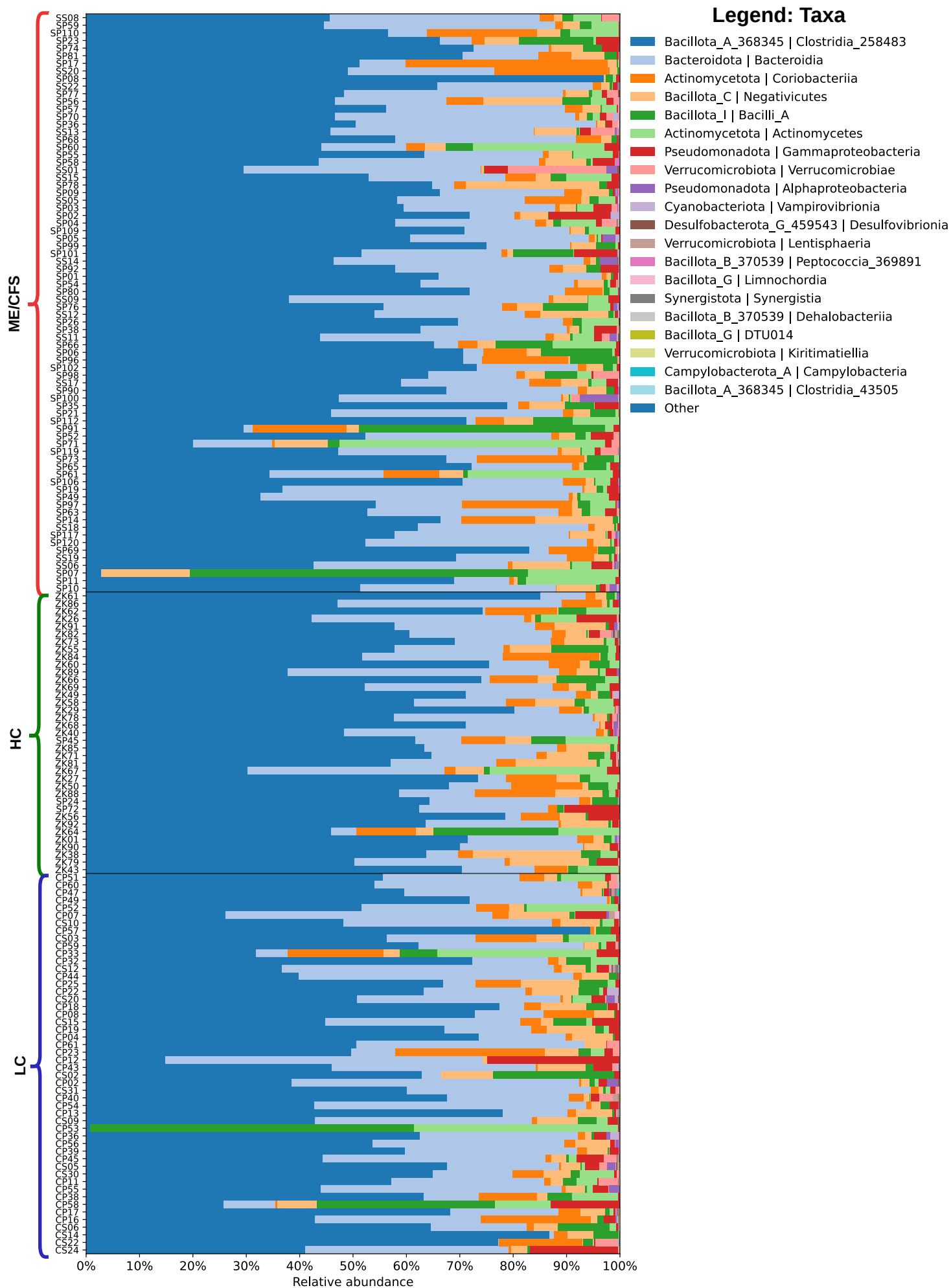

Supplement: Supplementary file 1 [file biomedicines-14-01183-s001.zip › suppl. files/Supplementary Figure S2. Phylum-level taxonomic composition.pdf]

**A**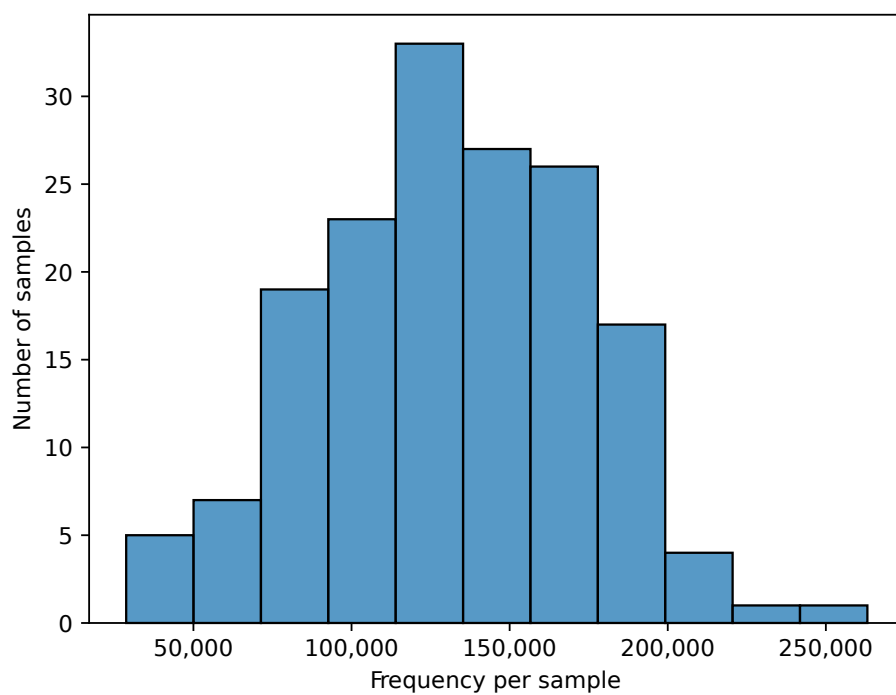**B**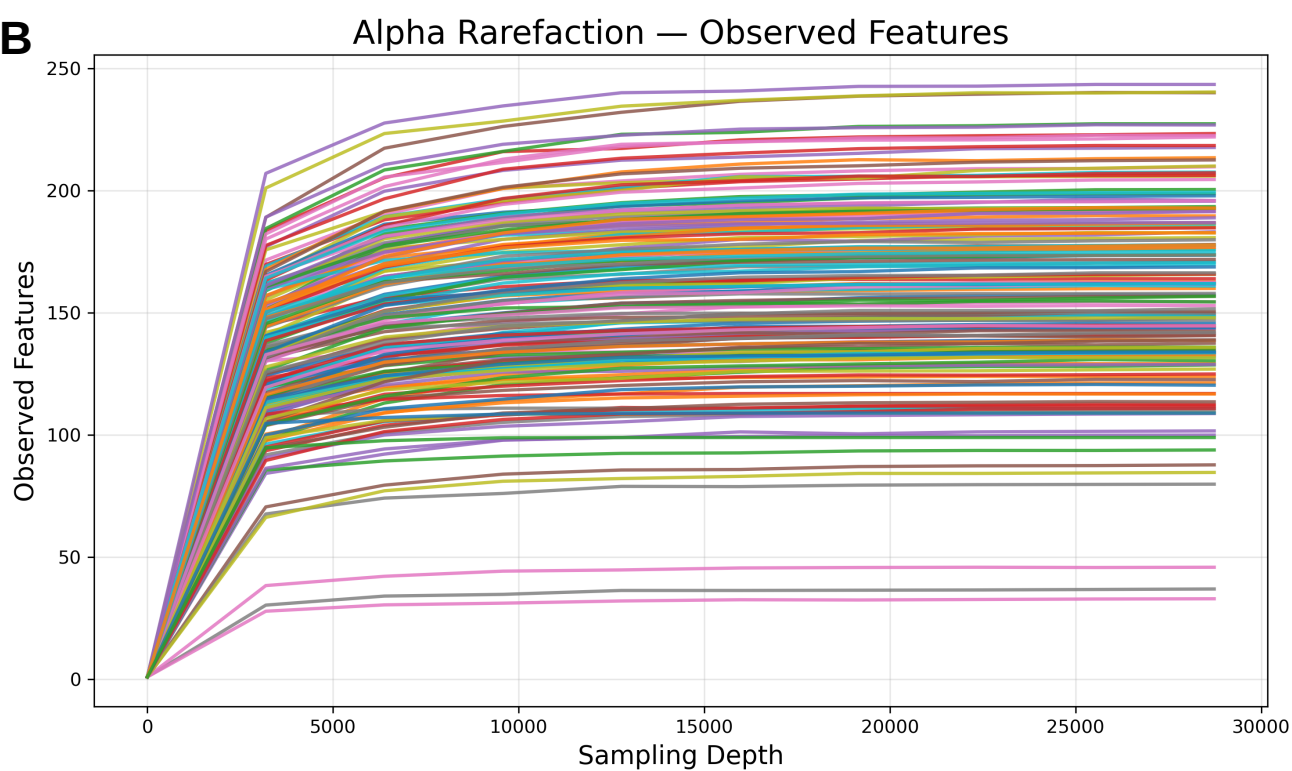

Supplement: Supplementary file 1 [file biomedicines-14-01183-s001.zip › suppl. files/Supplementary Figure S1. Sequencing depth and rarefaction overview.pdf]

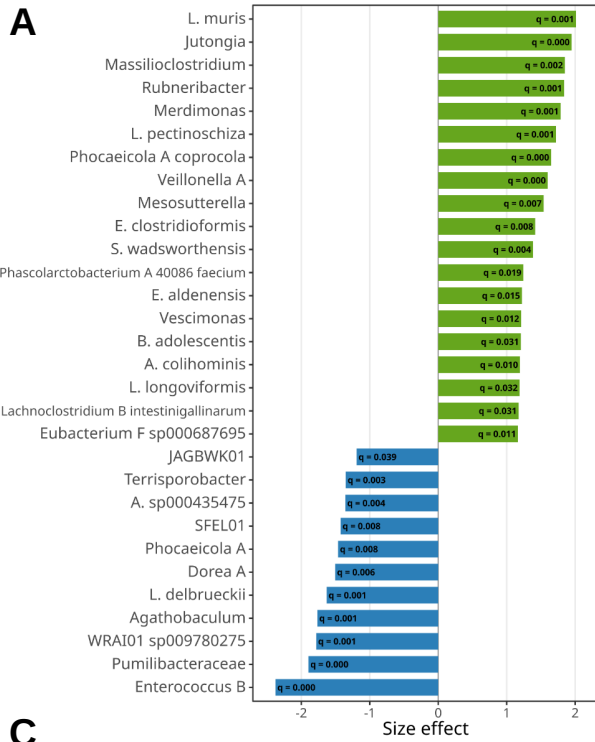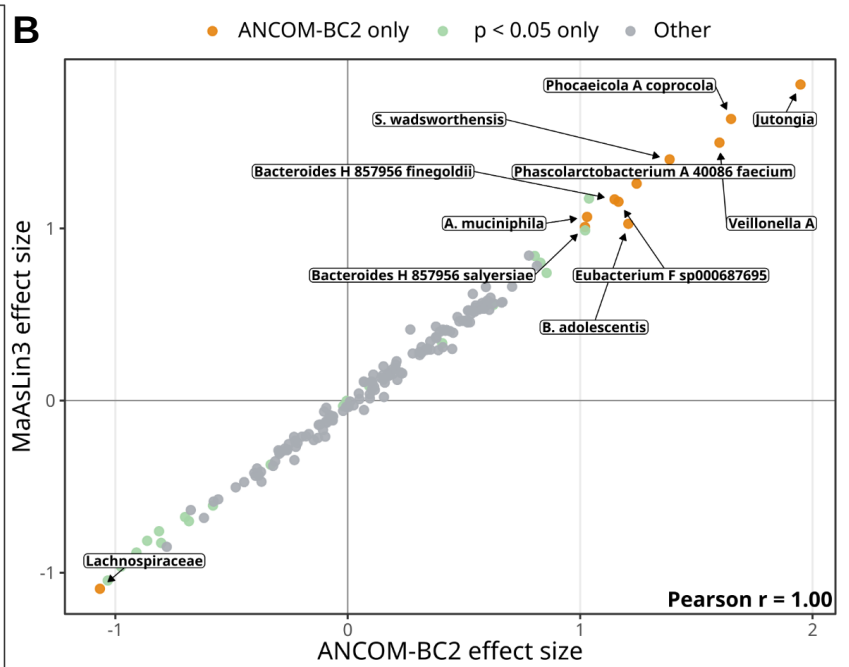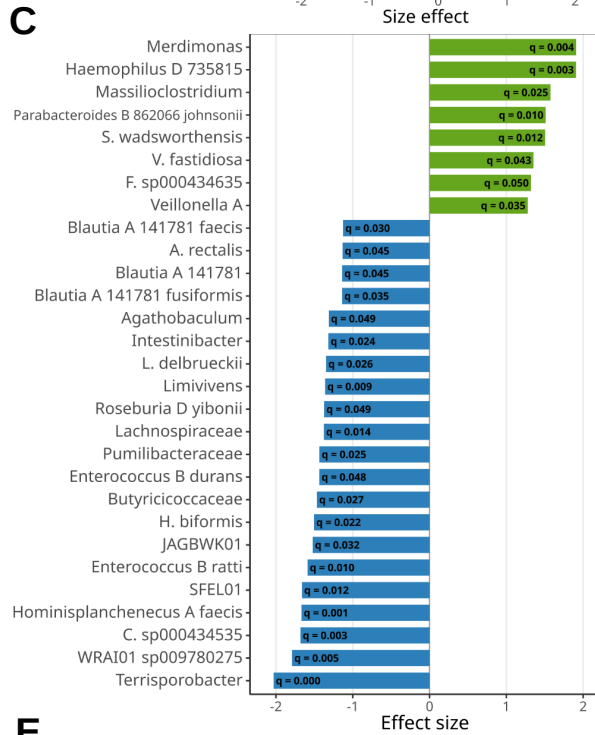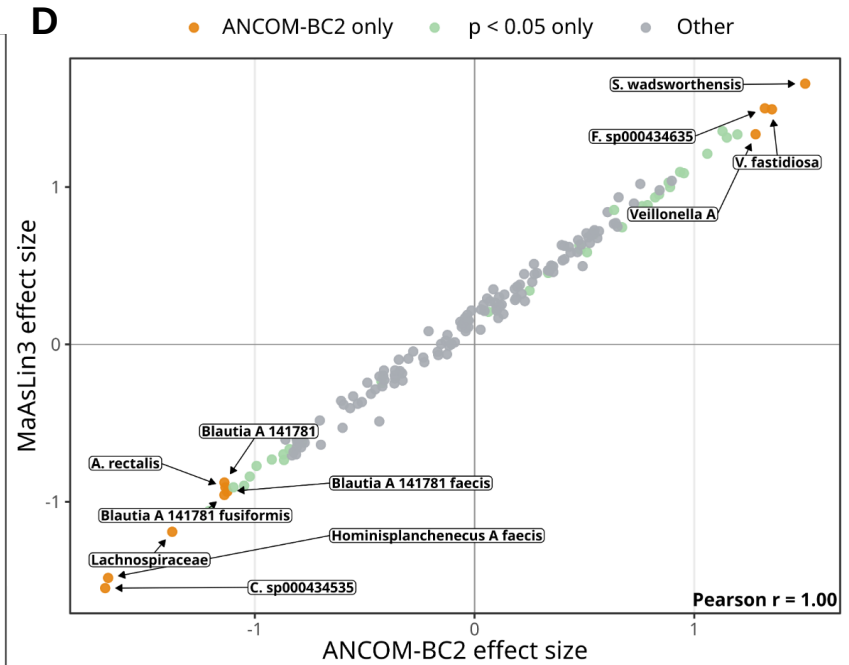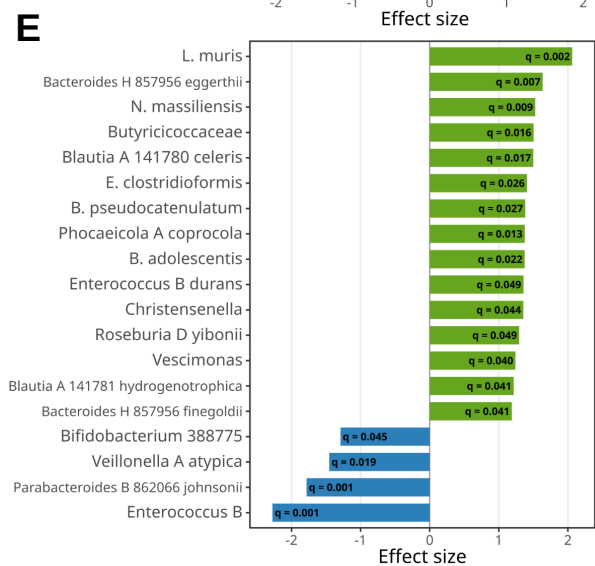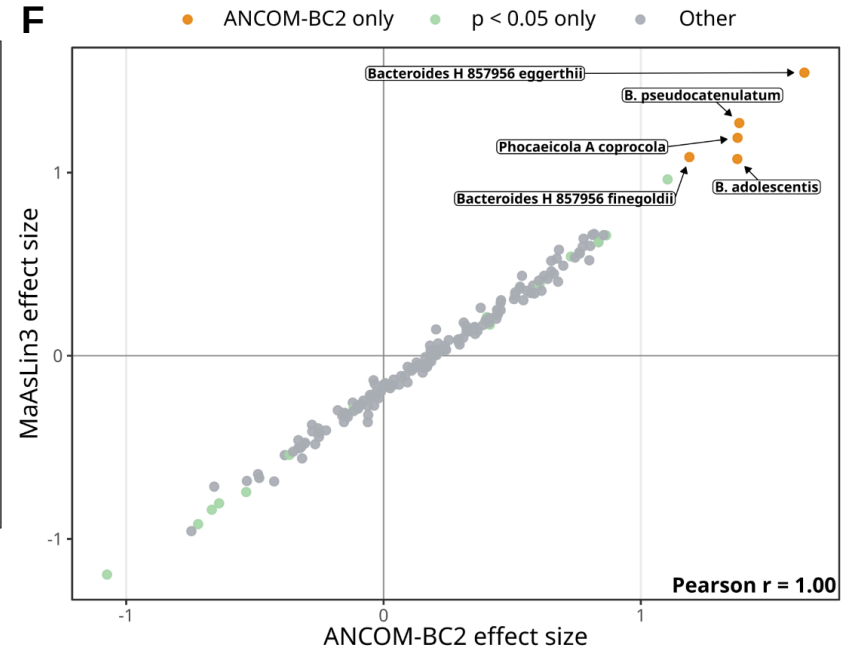

Supplement: Supplementary file 1 [file biomedicines-14-01183-s001.zip › suppl. files/Supplementary Figure S5. Species-level agreement between ANCOM-BC2 and MaAsLin3.pdf]

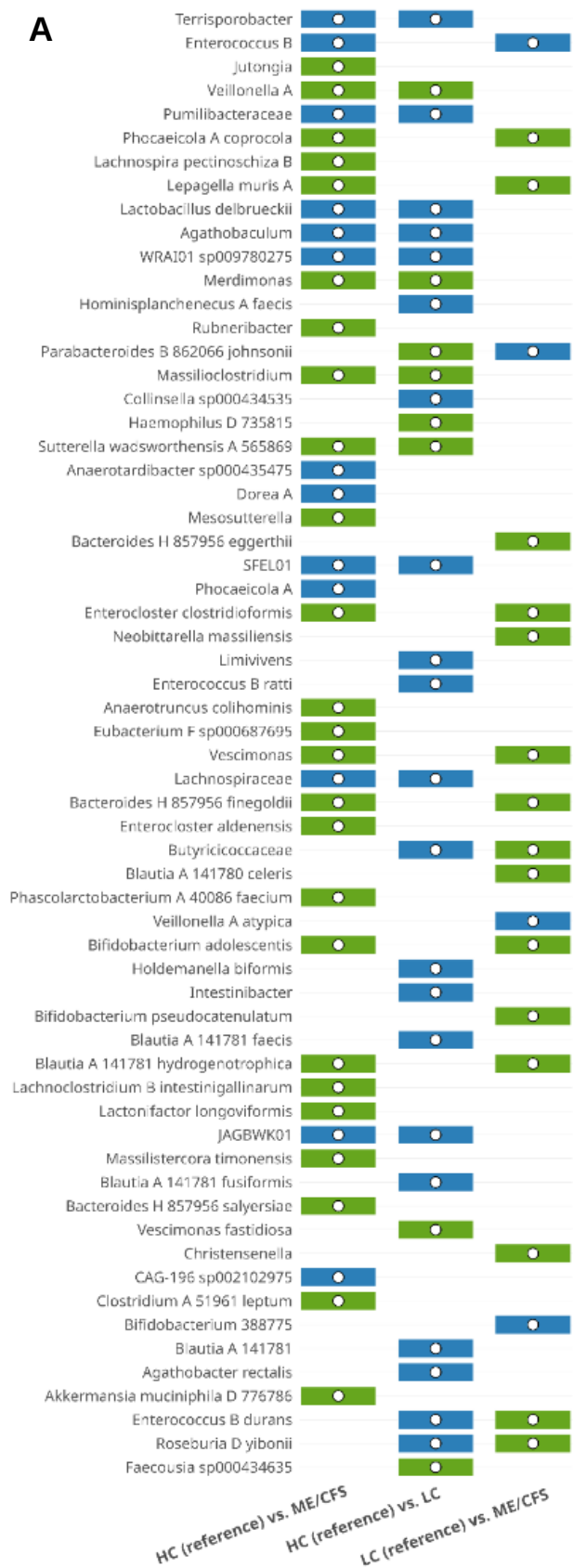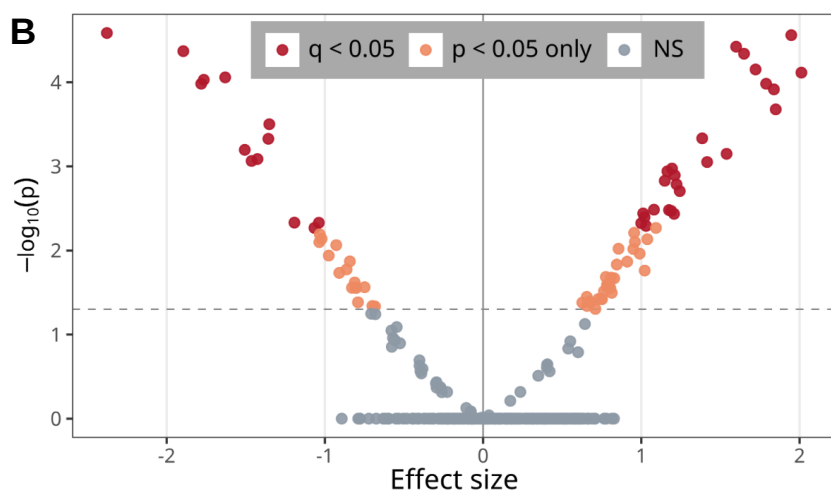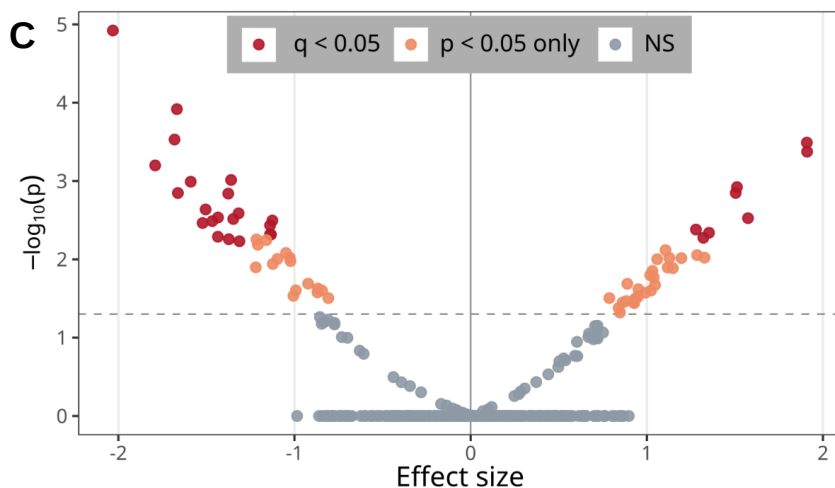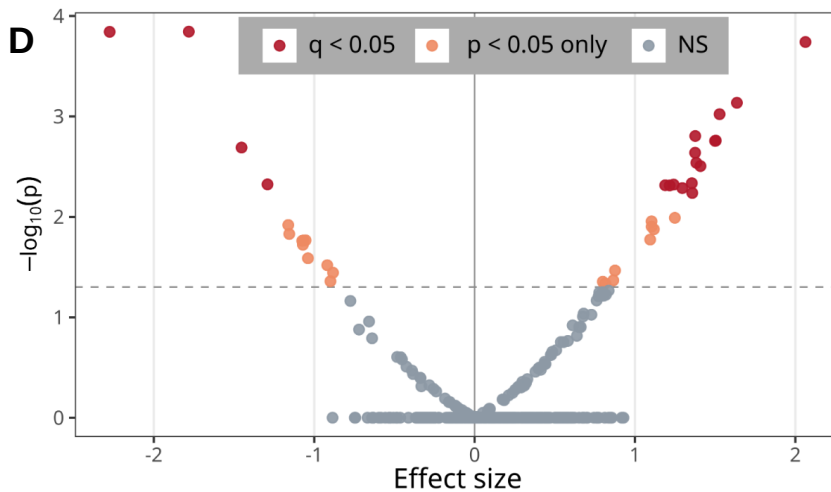

Supplement: Supplementary file 1 [file biomedicines-14-01183-s001.zip › suppl. files/Supplementary Figure S4. Species-level differential abundance results.pdf]
